# Exercise and cardiovascular health among breast cancer survivors: a scoping review of current observational evidence

**DOI:** 10.1186/s40959-025-00310-z

**Published:** 2025-02-26

**Authors:** Oliver W.A. Wilson, Kaitlyn M. Wojcik, Camryn M. Cohen, Dalya Kamil, Gisela Butera, Charles E. Matthews, Christina M. Dieli-Conwright, Jinani Jayasekera

**Affiliations:** 1 National Institute on Minority Health and Health Disparities, Intramural Research Program, National Institutes of Health, Bethesda, MD USA; 2 Clinical Genetics Branch, National Cancer Institute, National Institutes of Health, Bethesda, MD USA; 3 Office of Research Services, National Institutes of Health Library, Bethesda, MA USA; 4 Metabolic Epidemiology Branch, National Cancer Institute, National Institutes of Health, Bethesda, MD USA; 5 Division of Population Sciences, Dana-Farber Cancer Institute and Harvard Medical School, Boston, MA USA

**Keywords:** Physical activity, Resistance training, Cardiovascular disease cardiac function, Diabetes, Hypertension, Cholesterol, Kidney disease

## Abstract

**Background:**

Breast cancer survivors are at increased risk of cardiovascular events due to the cardiotoxic effects of cancer treatment. Exercise participation can lower the risk of various adverse cardiovascular health outcomes. However, most breast cancer survivors do not meet exercise guidelines.

**Objectives:**

To map and critically evaluate the observational literature describing the direction and strength of the relationship between post-diagnosis leisure-time exercise (aerobic and muscle-strengthening) and cardiovascular health (cardiovascular disease, cardiac function, and related physiological risk factors) among diverse breast cancer survivors; and identify variations in this relationship based on race, ethnicity, and/or socioeconomic status.

**Methods:**

Our scoping review was conducted in accordance with established guidelines and frameworks. Seven databases were searched. Participant characteristics, findings regarding the relationship between exercise and cardiovascular health, and any variations in this relationship were extracted. Article quality was appraised using the Mixed Methods Appraisal Tool.

**Results:**

Fourteen sources were identified, and study quality varied. Two adjusted analyses found aerobic exercise may lower the risk of cardiovascular disease. There was limited data found on the direction and strength of an adjusted relationship between exercise (aerobic or muscle-strengthening) and other cardiovascular outcomes or possible variations in the relationship across racial, ethnic, or socioeconomic groups.

**Conclusion:**

Findings highlight a considerable gap in knowledge regarding the relationship between exercise and cardiovascular health among diverse breast cancer survivors. Further longitudinal observational research is needed to better establish the direction and strength of this relationship, and how it differs based on race, ethnicity, or socioeconomic status.

**Supplementary Information:**

The online version contains supplementary material available at 10.1186/s40959-025-00310-z.

## Background

 Breast cancer survivors are at higher risk (9%) of cardiovascular disease-related mortality compared to cancer-free women [1]. Adverse (cardiotoxic) effects of cancer treatment [2–4], preexisting comorbid conditions [2], and reductions in exercise following cancer diagnosis [5–9] may each contribute to an increased risk of cardiovascular events among women diagnosed with breast cancer. Exercise has been shown to reduce the risk of cardiovascular disease [10–12], related mortality [11], and physiological risk factors, such as hypertension and type II diabetes [10, 12], within the general population.

However, many breast cancer survivors experience challenges in maintaining exercise levels during and following cancer treatment [5, 6]. For instance, fewer female breast cancer survivors in the U.S. meet aerobic (37.7%) or muscle-strengthening (17.6%) exercise guidelines compared to women without cancer (aerobic: 40.9%; muscle-strengthening: 18.6%) [13]. Importantly, studies report disparities in exercise participation [13–15] and cardiovascular outcomes among breast cancer survivors [16, 17]. For example, lower proportions of Black and Hispanic breast cancer survivors meet exercise guidelines compared to survivors overall [13], and heart disease mortality is higher among Black and Hispanic breast cancer survivors compared to White survivors [17]. However, there is limited evidence summarizing the possible variations in the relationship of exercise with cardiovascular outcomes across race, ethnicity, and socioeconomic characteristics among breast cancer survivors. This information may help develop individualized exercise prescriptions [18–20] to increase exercise participation, improve cardiovascular health, and reduce disparities among breast cancer survivors.

Findings from exercise interventions show that exercise can lower the risk of cardiovascular disease and improve cardiopulmonary function of breast cancer survivors [21–23]. However, these interventions often include extensive supervision and/or behavioral support which may not be feasible or accessible to breast cancer survivors outside a clinical trial or an intervention study [24]. Furthermore, due to lack of diversity, the findings from studies may also show limited generalizability for underrepresented and underserved groups [25, 26]. In this context, observational studies are useful in evaluating the effects of exercise in real-world settings [24]. Therefore, in this review, we focused on the observational literature to summarize the relationship between exercise and cardiovascular health among diverse breast cancer survivors in real-world settings.

At present, there are currently gaps in knowledge regarding the direction and strength of the real-world relationship between exercise and cardiovascular health among breast cancer survivors, as well as how this relationship differs based on race, ethnicity, and socioeconomic status. Therefore, the overarching goal of this scoping review was to map and critically evaluate the observational literature describing the direction and strength of the relationship between post-diagnosis leisure-time exercise (aerobic and muscle-strengthening) and cardiovascular health (cardiovascular disease, cardiac function, and related physiological risk factors) among diverse breast cancer survivors. In a secondary aim, we evaluated the differences in the relationship between exercise and cardiovascular health based on racial, ethnic, and socioeconomic characteristics.

## Methods

We conducted a scoping review using the methodological framework established by Arksey & O’Malley and further refined by Joanna Briggs Institute methodology [27–29]. The review followed the Preferred Reporting Items for Systematic reviews and Meta-Analyses extension for Scoping Reviews (PRISMA-ScR) [30] (see Supplement 1for the PRISMA-ScR Checklist [30]). The review protocol was registered in Open Science Framework [https://osf.io/tfnuq/?view_only=92beda9c9567439c8bf3e2ce4b66fc82].

### Definitions

#### Breast cancer survivors

In accordance with the American Cancer Society (ACS) definition, the population of interest for this review included individuals ever diagnosed with breast cancer, regardless of where they are in the course of the disease [31]. The consideration of individuals ever diagnosed with breast cancer in this study allowed us to evaluate the possible variations in the relationship between exercise and cardiovascular health across demographic (e.g., race) and socioeconomic characteristics of breast cancer survivors.

#### Exercise

Subjective physical activity measures collect information about physical activity participation in one or more of the following domains: leisure (i.e., recreational), occupational, household, and transportation [32]. Most physical activity measures would typically collect information on leisure-time exercise as it is considered the most modifiable form of exercise [19]. Further, epidemiological research has traditionally focused on leisure-time exercise [32]. Thus, for this review, leisure-time exercise (aerobic and muscle-strengthening) was selected to facilitate cross-study comparisons.

#### Cardiovascular health

The primary outcome of interest was cardiovascular health, which included cardiovascular disease (i.e., heart disease, heart attack, stroke, and heart failure), cardiac function (e.g., ventricular function), and related physiological risk factors (i.e., diabetes, hypertension, cholesterol, and kidney disease) according to the American Heart Association and Centers for Disease Control and Prevention [2, 33, 34].

### Data sources and search strategy

We conducted a search of the published literature that reported on the association of exercise with recurrence, mortality, and/or quality of life among breast cancer survivors (Supplement 2). The search strategy was developed using an iterative approach by a trained librarian (GB) at the National Institutes of Health and peer reviewed. The search strategy included a combination of keywords, synonyms, Medical Subject Headings (MeSH) terms, and Emtree terms. The search was originally performed on September 18, 2023, across seven widely used scientific databases: MEDLINE via PubMed (National Library of Medicine), PsycINFO (American Psychological Association), Embase (Elsevier), Scopus (Elsevier), Web of Science Core Collection (Clarivate Analytics), Cochrane CENTRAL (Wiley & Sons), and CINAHL Plus (EBSCO). The search was updated on October 4, 2024. Database date searches were limited from 01/2003 onwards with English-only language restrictions. In addition to sources identified via databases, we also searched the prior and derivative works of all sources that proceeded to extraction.

### Inclusion and exclusion criteria

Inclusion and exclusion criteria are detailed in Supplement 3. Inclusion criteria were: (I) breast cancer survivors; (II) examination of the association of exercise with cardiovascular health; (III) empirical observational research studies; and (IV) full-text available in English.

### Screening procedure

Screening was conducted in Covidence screening software (Veritas Health Innovation, Melbourne, Australia) [35]. Sources from database searches were first imported into EndNote 21 (Clarivate) to remove duplicates and then imported into Covidence to identify additional duplicates. Screening of titles, abstracts, and full texts were conducted independently by four authors (OW, KW, CC, DK), with discrepancies resolved through discussion.

### Quality appraisal

Article quality was appraised using the two screening questions and four items relating to quantitative non-randomized research from the Mixed Methods Appraisal Tool [36]. Items pertain to sample representativeness, measure appropriateness, data completeness, and whether confounders were accounted for. We set the threshold for data completeness at 80% [37] and focused on appraising methods used to assess effect of exercise on cardiovascular health.

### Data charting

Data charting was conducted independently by four authors (OW, KW, CC, DK) using a data extraction form within Excel. Information was extracted on study location (country); data source; study purpose; research design; number of participants; individual (e.g., age) and clinical (e.g., stage) characteristics; race and ethnicity; contextual characteristics (e.g., socioeconomic status); exercise measurement methods and proportion meeting guidelines; relevant findings; study limitations; funding source; and conflicts of interest.

### Data synthesis

Data were analyzed and summarized descriptively using a narrative approach supported by accompanying tables.

### Uncertainty

There are severe limitations to narrative synthesis of results based on statistical significance [38]. Since evidence lies on a continuum, presenting findings as significant vs. non-significant would reduce the value of the insights available in the reported data [39, 40]. As such, synthesis focused on the direction and strength of relationships as opposed to binary significance testing [41].

## Results

**Fig. 1:**
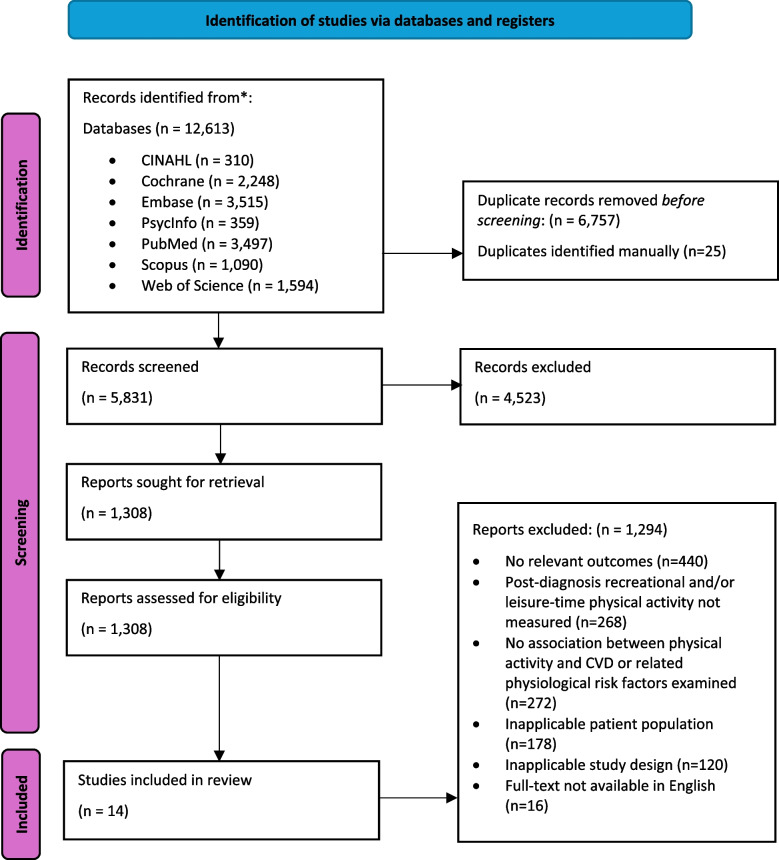
Article identification process using research framework

Initial searches retrieved 5,831 sources after the removal of duplicates. These were screened at the title and abstract levels, followed by a full-text review of 1,308 remaining sources. No additional articles were identified from searching reference lists. Ultimately, 14 studies were identified and proceeded to extraction (Fig. 1).

### Source characteristics

Study and participant characteristics are reported in Tables 1 and 2. Six studies originated from the U.S. [42–47], two from South Korea [48, 49], and others from Canada [50], China [51], Germany [52], Ireland [53], Spain [54], and Sweden [55]. Most studies included women who predominantly had stage 0 through II breast cancer, were aged > 50 years, and had received some form of treatment. Time since diagnosis varied considerably from only six months [51] to an average of more than five years [52]. Details on the race/ethnicity of participants in the studies from Canada, Germany, Spain, and Sweden were either not reported or collected [52, 54, 55]. The studies from China and Korea included predominantly Asian women [48, 49, 51]. Among U.S. studies, one focused on Black women [46], whereas the remainder included predominantly White women. Most women had at least a high school education within studies that reported education. The location (e.g., rural vs. urban) was not specified by any study.

No stu﻿dies reported sub-group analyses based on racial, ethnic, or socioeconomic characteristics. Heterogeneity in the classification and use of the exposure variable (i.e., exercise) within analyses, measurement and classification of outcome variables, and analytical approaches limited cross-study comparisons. As such, information on participant characteristics and main study findings were extracted and reported for total samples.

**Table 1:** Study characteristics – clinical and biological characteristics

Author	Location (Data Source)	Study Design (Sample Size)	Gender	Stage	Age (Years)	Treatment	Time Since diagnosis (Years)
Peck et al. 2022 [50]	Canada (EMBRACE-MRI 1)	Prospective cohort (*n* = 88)	Women only	I: 9% II: 61% III: 30% IV: 1%	M = 51.4 (SD = 8.9)	Undergoing CT	-
Bao et al. 2013 [51]	China (SBCSS)	Prospective cohort (*n* = 1696)	Women only	0-I: 41.6% IIb 48.7% III: 5.1%	M = 51.4 (SD = 7.8)	CT: 93.9% HT (Tamoxifen): 54.5% RT: 30.7%	0.5
Obi et al. 2014 [52]	Germany (MARIE)	Prospective cohort (*n* = 2,542)	Women only	In situ: 6.4% I: 45.7% II:36.0% III/IV: 8.8%	M = 62.1 (SD = 5.9)	CT: 45.8% HT: Tamoxifen (68.9%), AI (46.2%), Trastuzumab (68.9%) RT: 78.7%	M = 5.8
Guinan et al. 2013 [53]	Ireland (Oncology clinics)	Cross-sectional (*n* = 69)	Women only	I: 27.5% II: 52.2% III: 20.3%	M = 53.4 (SD = 9.4)	CT: 87.0% HT: Tamoxifen (50.7%), AI (29.0%), Herceptin (11.6%) RT: 81.2% Surg (Mastectomy): (55.1%)	Must have completed adjuvant treatment
Kim et al. 2021 [48]	South Korea (Korea NHIS)	Prospective cohort (*n* = 39,775)	Women only	Not collected	M = 51.2 (SD = 9.3)	CT (Cardiotoxic): 45.2% HT: Tamoxifen (5.1%), AI: (8.6%) RT: 58.0%	≥ 5
Kim, So, & Kim, 2020 [49]	South Korea (KHANES)	Retrospective Cohort (*n* = 187)	Women only	-	M = 54.5 (SD = 11.3)	-	~M = 5
Ariza-García et al. 2013 [54]	Spain (Oncology department)	Cross-sectional (*n* = 108)	Women only	I: 38.0% II: 25.0% IIIa: 40.0%	M = 49.18 (SD = 8.25)	CT only: 5.6% RT only: 6.5% CT + RT: 88.0% Surg (Mastectomy): 31.5%	≥ 1-month post-primary oncology
Nilsson et al. 2016 [55]	Sweden (Hospitals)	Prospective cohort (*n* = 220)	Women (99.5%) scheduled to have BC surgery	Not collected	M = 60.5 (SD = 11.9)	Surg (Mastectomy): 28%	-
Busen et al. 2023 [46]	U.S. - GA, TN, SC (AABL)	Cross-sectional (*n* = 323)	Women only	0: 13.2% I: 38.4% II: 35.5% III/IV: 12.9%	M = 59.1 (range 27.9–79.5)	CT: 57.0% HT (AI): 32.0% RT: 66.8% Surg (Mastectomy): 39.2%	-
Upshaw et al. 2020 [47]	U.S. - PA (CCT cohort)	Prospective cohort (*n* = 603)	Men and women who planned CT	I: 22.0% II: 54.3% III: 22.2% IV: 1.5%	Md = 50 (IQR = 42, 58)	RT: 64.9%	Soon after diagnosis
Irwin et al. 2005 [42]	U.S. - CA, NM, WA (HEAL)	Prospective cohort (*n* = 710)	Women only	0: 24% I: 55% II-IIIA: 21%	M = 55.1 (SD = 10.2)	CT: 30% Surg only: 31% RT + Surg: 39%	-
Jones et al. 2016 [43]	U.S. - CA, UT (LACE + Pathways)	Prospective cohort (*n* = 2,973)	Women only	I: 49.8% II: 42.9% III: 7.4%	M = 58.0 (SD = 10.5)	CT: 55.9% HT (Tamoxifen or AI): 75.3% RT: 52.3% Surg (Mastectomy): 42.3%	-
Marell et al. 2023 [44]	U.S. - MN (Breast Disease Registry)	Prospective cohort (*n* = 171)	Women (98.8%)	Non- metastatic	< 50: 24.5% ≥ 50: 74.4%	CT: 29.2% HT: 58.5% RT: 55.0% Surg (Mastectomy): 51.4%	< 1
Dieli-Conwright et al. 2022 [45]	U.S. (WHI)	Prospective cohort (*n* = 8543)	Post-menopausal women only with anticipated 3-year survival	Localized: 76.8% Regional: 23.2%	50–59: 34.5% 60–79: 61.9%	CT: ~29% HT (or antiestrogen pills):~68% RT:~70%	-

Notes. *AABL* African American Breast Cancer Long-Term Survivorship, *CCT* Cardiotoxicity of Cancer Therapy, *EMBRACE-MRI 1* Evaluation of Myocardial Changes During Breast Adenocarcinoma Therapy to Detect Cardiotoxicity Earlier With MRI, *HEAL* Health, Eating, Activity, and Lifestyle, *KHANES* Korea National Health and Nutrition Examination Survey, *LACE* Life After Cancer Epidemiology, *MARIE* Mamma carcinoma Risk factor Investigation, *NHIS* National Health Insurance Service, *SBCSS* Shanghai Breast Cancer Survival Study, *WHI* Women’s Health Initiative, *BC* Breast Cancer, *M* Mean, *SD* Standard Deviation, *Md* Median, *CT* Chemotherapy, *HT* Hormone (endocrine) Therapy, *RT* Radiation Therapy, *IT* Immunotherapy, *AI* Aromatase Inhibitors, *Surg* Surgery

**Table 2:** Study characteristics - Race/ethnicity, education, and income

**Author**	**Location (Data Source)**	**Race/Ethnicity**					**Education**				**Annual Income (USD)**		
		**Asian**	**Black**	**Hispanic**	**White**	**Other**	**≤HS**	**HS/GED**	**Some College**	**≥ College**			
Peck et al. 2022 [50]	Canada (EMBRACE-MRI 1)	Not reported					Not reported				Not reported		
Bao et al. 2013 [51]	China (SBCSS)	Predominantly Chinese	-	-	-	-	42.8%	43.2%	.	14.1%	-	14.1%	NR
Obi et al. 2014 [52]	Germany (MARIE)	Not reported					Low: 55.3%; Medium: 28.8%; High: 15.9%				-		
Guinan et al. 2013 [53]	Ireland (Oncology clinics)	-	-	-	100% (Irish)	-	Not collected				Not collected		
Kim et al. 2021 [48]	South Korea (Korea NHIS)	Predominantly Korean	-	-	-	-	Not reported				≥Md: 45.7% <Md: 54.3%		
Kim, So, & Kim, 2020 [49]	South Korea (KHANES)	Predominantly Korean	-	-	-	-	Not reported				Not reported		
Ariza-García et al. 2013 [54]	Spain (Oncology department)	Not reported					35.2%	25.0%	-	39.8%	Not reported		
Nilsson et al. 2016 [55]	Sweden (Hospitals)	Not collected					Not available						
Busen et al. 2023 [46]	U.S. - GA, TN, SC (AABL)	-	100%	-	-	-	28.0%	-	36.3%	35.7%	<$50K: 64.0% ≥$50K: 35.6%		
Upshaw et al. 2020[47]	U.S. - PA (CCT cohort)	.	25.2%	2.3%	70.4%	4.3%	Not reported				Not reported		
Irwin et al. 2005 [42]	U.S. - CA, NM, WA(HEAL)	-	24.6%	10.6%	64.8%	-	-	94%	-	-	Not reported		
Jones et al. 2016 [43]	LACE + Pathways	-	-	-	72.3%	27.7%	Not reported						
Marell et al. 2023 [44]	U.S. - MN (Breast Disease Registry)	-	1.2%	1.8%	95.5%	Other:2.9%	-	11.1%	31%	57.9%	Not reported		
Dieli-Conwright et al. 2022 [45]	U.S. (WHI)	Asian/Pacific Islander: 2.3%	6.0%	2.3	89.1%	American Indian:0.4%	17.9%	≥HS/GED: 82.1%			Not reported		

Notes. *NR* Not reported, *HS* High school, *GED* General educational development, *USD *U.S. dollars, *AABL* African American Breast Cancer Long-Term Survivorship, *CCT* Cardiotoxicity of Cancer Therapy, *EMBRACE*-*MRI 1* Evaluation of Myocardial Changes During Breast Adenocarcinoma Therapy to Detect Cardiotoxicity Earlier With MRI, *HEAL* Health, Eating, Activity, and Lifestyle, *KHANES* Korea National Health and Nutrition Examination Survey, *LACE* Life After Cancer Epidemiology, *MARIE* Mamma carcinoma Risk factor Investigation, *NHIS *National Health Insurance Survey, *SBCSS* Shanghai Breast Cancer Survival Study, *WHI* Women’s Health Initiative, *Md* Median

### Quality appraisal

All studies had a clear research question, collected appropriate data to answer research questions, and included participants that were representative of the target population. All but three used appropriate measures, and all but five had complete outcome data. Eight accounted for confounders in design or analyses (see Supplement 4).

### Aerobic exercise

#### Measurement and exercise guidelines

Three studies used the Godin Leisure Time Exercise Questionnaire [44, 47, 50], two used recreational dimension of the Arizona Activity Frequency Questionnaire [43, 46], two used the used the Minnesota Leisure Time Physical Activity Questionnaire (PAQ) [53, 54], and two used unnamed measures of recreational exercise [49, 52]. Single studies used recreational items from the International PAQ-Long Form [48], the Modifiable Activity Questionnaire [56], Saltin-Grimby Physical Activity Level Scale [55], Shanghai Women’s Health Study PAQ [51], and recreational items from the Women’s Health Initiative PAQ [45],

Eight studies [43–49, 53] reported the proportion of participants meeting the equivalent of exercise guidelines (≥ ~150 min/week) from various organizations (e.g., ACS) [18, 19, 57–59]. The average proportion meeting guidelines was 51.0% (*n* = 52,644). However, Kim et al. 2021 [48], who had a sample of 39,775, of whom 53.3% met guidelines, skewed this average. Excluding Kim et al. 2021 [48], the average was 43.9% (*n* = 12,869) (Supplement 5) [43–47, 49, 53].

#### Cardiovascular diseases

Three studies reported adjusted Cox proportional hazard regression analyses to quantify the longitudinal association between exercise and cardiovascular diseases (Table 3). However, we were unable to compare studies due to differences across referent and comparison groups. Jones et al. 2016 [43], which included a sample of predominantly White women, found that meeting exercise guidelines was associated with lower risk of cardiovascular events (HR:0.77; 95% CI:0.67–0.88), coronary artery disease (HR:0.74; 95% CI:0.55–0.99), and heart failure (HR:0.71; 95% CI:0.56–0.90) [43]. They also reported a series of adjusted hazard ratios for cardiovascular events according to meeting (≥ 9 MET-hrs/week) vs. not meeting (< 9 MET-hrs/week) exercise guidelines in subgroups defined by age, menopausal status, physiological risk factors, and treatments. However, only treatment subgroups results included precision details [43]. Accordingly, among women receiving endocrine therapy, meeting guidelines lowered the risk of events by 22% among those who received aromatase inhibitors (HR:0.78; 95% CI:0.64–0.95) and 22% among those who did not (HR:0.78; 95% CI:0.64–0.94). For women undergoing chemotherapy, meeting guidelines lowered the risk of events by 23% among those who received doxorubicin-containing chemotherapy (HR:0.77; 95% CI:0.610.97) and 21% among those who did not (HR:0.79; 95% CI:0.66–0.94).

A study comprised of Korean women found that increasing levels of exercise above inactivity were associated with lower risk of cardiovascular disease (hospitalization for coronary heart disease or stroke for ≥ 2days), coronary artery disease, and heart failure (Table 3) [48]. Differences in comparison groups used by Obi et al. 2014 [52] limited our ability to draw conclusions regarding the magnitude, direction, and precision of the association between exercise and cardiovascular disease (angina pectoris, myocardial infraction, stroke, arterial occlusion disease) [52].

**Table 3:** Adjusted associations between exercise and cardiovascular diseases

Study	Outcomes	Hazard Ratios (95%CI)									
		MET-hrs/week									
		0	> 0-<2	> 0-~8.3	< 9	>~8.3-~16.7	≥ 9	>~16.7	< 24	< 42	≥ 42
Jones et al. 2016 [43]	Cardiovascular events	-	-	-	Referent	-	0.77 (0.67–0.88)	-	-	-	-
	Coronary artery disease	-	-	-	Referent	-	0.74 (0.55–0.99)	-	-	-	-
	Heart failure	-	-	-	Referent	-	0.71 (0.56–0.90)	-	-	-	-
Kim et al. 2021 [48]	Cardiovascular disease^b^	Referent	-	0.83 (0.69–0.99)	-	0.77 (0.64–0.93)	-	0.73 (0.60–0.89)	-	-	-
	Coronary artery disease^b^	Referent	-	0.90 (0.68–1.19)	-	0.79 (0.59–1.06)	-	0.75 (0.55–1.03)	-	-	-
	Heart failure^b^	Referent	-	0.78 (0.62–0.98)	-	0.76 (0.60–0.96)	-	0.72 (0.56–0.93)	-	-	-
Obi et al. 2014 [52]	Cardiovascular disease^a+physician confirmed^	Referent	0.81 (0.55–1.20)	-	-	-	-	-	1.08 (0.73–1.58)	0.84 (0.55–1.27)	0.57 (0.36–0.91)

All analyses adjusted for age, BMI, smoking, adjuvant therapies, diabetes or glucose; Jones et al. 2016 also adjusted for race, menopausal status, stage, study, and pre-existing peripheral artery disease and/or hyperlipidemia and/or hypertension; Kim et al. 2021 also adjusted for Adjusted for, household income, alcohol, systolic blood pressure, total cholesterol, Charlson comorbidity index, and diagnosis year; Obi et al. 2014 also adjusted for study region, postmenopausal at breast cancer diagnosis, marital status, education, age at menarche, menopausal hormone therapy at breast cancer diagnosis, parity and alcohol consumption, tumor stage, a combined variable of her2/neu receptor status

*MET* Metabolic equivalent

^a^Self-report

^b^International Classification of Diseases

^-^ = data not reported

Using unadjusted cross-sectional analyses, five studies compared the proportion of participants with a range of cardiovascular diseases between those who did and did not meet exercise guidelines (Table 4). One study including only Black women found that the proportion with heart disease or to have experienced a stroke was considerably lower among those who met guidelines [46]. Similarly, two studies of predominantly White women found that the proportion with heart failure [43], heart disease [44], coronary artery disease [43], or to have experienced a cardiovascular event [43] or a stroke [44] were considerably lower among those who met guidelines, whereas another found no difference in cardiovascular death between those who did and did not meet guidelines among predominantly White women [45]. No differences were observed among Korean women, though the prevalence of cardiovascular diseases was very low [48].

**Table 4:** Unadjusted associations between exercise and cardiovascular diseases

**Study**	**Outcomes**	**Exercise**		
		**Guidelines**	**Met Guidelines**	
			**Yes**	**No**
			**%**	
Busen et al. 2023 [46]	Heart disease^a^	≥150 min/week of MPA or ≥75 min/week of VPA (ACS/USDHHS)	11.7	18.0
	Stroke^a^		0.8	7.2
Dieli-Conwright et al. 2022 [45]	Cardiovascular death	≥9 MET-hrs/week	7.3	7.9
Jones et al. 2016 [43]	Cardiovascular events	≥9 MET-hrs/week	24.0	33.9
	Coronary artery disease		5.4	8.3
	Heart failure		7.9	12.8
	Peripheral vascular disease		0.9	1.2
Kim et al. 2021 [48]	Cardiovascular events^b^	>500 MET-min/week	1.9	2.7
	Coronary heart disease^b^		0.7	1.1
	Stroke^b^		1.2	1.6
Marell et al. 2023 [44]	Heart disease^a^	≥150 min/week of MVPA (ACSM, USDHHS, NCCN)	4.6	5.9
	Stroke^a^		0.0	2.9

*MET* Metabolic equivalent, *MPA* moderate-intensity physical activity, *VPA* vigorous-intensity physical activity, *ACS* American Cancer Society, *USDHHS* U.S. Department of Health and Human Services, *ACSM* American College of Sports Medicine, *NCCN* National Comprehensive Cancer Network

^a^Self-report

^b^International Classification of Diseases

#### Cardiac function

Two studies examined the association of exercise with indicators of cardiac function. The study by Upshaw et al. 2020 involving a sample of White women found, using adjusted longitudinal analyses, that higher exercise was associated with a modest improvement in the absolute value of a left ventricular ejection fraction (β:0.36%, 95% CI:0.06–0.66 for each 10-unit increase in exercise), which approximates a change from sedentary to mild activity or from mild activity to moderate activity [47]. The other, presumably involving predominantly White women, found exercise was associated with better diastolic and systolic left ventricular function measures during treatment using adjusted analyses [50].

#### Diabetes

Eight studies reported findings concerning diabetes-related outcomes, two of which reported adjusted cross-sectional analyses (Table 5). Bao et al. 2013 found that exercise was modestly associated with higher fasting plasma glucose (≥ 5.6 mmol/L) among Chinese women, though data were statistically consistent with parameter values ranging from little to no effect to higher odds [51]. Another study including a relatively representative sample of women in the U.S. found that C-peptide, Leptin, and Insulin-Life Growth Factors were more favorable among women participating in more than minimal exercise [42].

Seven studies reported unadjusted cross-sectional analyses (Table 5). Five compared the unadjusted proportion of women with diabetes between those who did and did not meet exercise guidelines. One included only Black women [46], another predominantly Korean women [49], while the other three included predominantly White women [43–45]. Each of these studies reported a lower prevalence of diabetes among those who met guidelines. A study of White Irish women found that the prevalence of insulin resistance was considerably lower among those who met guidelines [53]. A study of Korean women found no difference in the fasting serum plasma glucose between those who did and did not meet guidelines [48].

**Table 5:** Associations of exercise with diabetes related outcomes

Study	Diabetes Outcome	Findings		
**Adjusted analyses**				
Bao et al. 2013 [51]^b^	Fasting plasma glucose (≥ 5.6 mmol/l)	Referent: No exercise < 3.5 hrs/week: OR = 1.27 (95% CI: 0.88, 1.83) ≥ 3.5 hrs/week: OR = 1.01 (95% CI: 0.70, 1.46)		
Irwin et al. 2005 [42]^c^		MET-hrs/week		
		< 2.6	2.6–13.2	> 13.3
		M (SD)		
	C-peptide (ng/mL)	2.5 (0.1)	2.4 (0.1)	2.0 (0.1)
	Leptin (ng/mL)	30.0 (1.1)	24.5 (1.1)	19.4 (1.1)
	IGF-I (ng/mL)	125.8 (3.4)	134.9 (3.3)	140.0 (3.4)
	IGFBP-3 (µg/mL)	4.0 (0.1)	4.1 (0.1)	4.2 (0.1)
	IGF-I: IGFBP-3	31.3 (0.7)	33.3 (0.7)	33.6 (0.7)
**Unadjusted analyses**				
		**Exercise**		
		**Guidelines**	**Met guidelines**	
			**Yes**	**No**
Busen et al. 2023 [46]	Diabetes^a^	≥ 150 min/week of MPA or ≥ 75 min/week of VPA (ACS/USDHHS)	20.3%	29.7%
Dieli-Conwright et al. 2022 [45]	History of diabetes^a^	≥ 9 MET-hrs/week	3.4%	5.7%
Guinan et al. 2013 [53]	Insulin resistant	≥ 30 min of MPA, ≥ 5 days/week (ACSM)	12.5%	30.2%
Jones et al. 2016 [43]	Type II diabetes	≥ 9 MET-hrs/week	6.6%	10.7%
Kim et al. 2021 [48]	Fasting serum plasma glucose (mg/dL)	> 500 MET-min/week	96.8 (19.4)	97.8 (21.1)
Kim, So, & Kim, 2020 [49]	Diabetes	≥ 150 min/week of MPA or ≥ 75 min/week of VPA	11.0%	15.5%
Marell et al. 2023 [44]	Diabetes^a^	≥ 150 min/week of MVPA (ACSM, USDHHS, NCCN)	1.5%	17.6%

Findings from only Irwin et al. 2005 [42] were included, while similar findings from three other HEAL study articles [60–62] were excluded, as it had the largest sample

*OR* Odds ratio, *MET* Metabolic equivalent, *MPA* moderate-intensity physical activity, *VPA* vigorous-intensity physical activity, *MVPA* moderate-to-vigorous-intensity physical activity, *ACS* American Cancer Society, *USDHHS* U.S. Department of Health and Human Services, *ACSM* American College of Sports Medicine, *NCCN* National Comprehensive Cancer Network, *IGF-I* Insulin-like growth factor 1, *IGFBP-3* Insulin-like growth factor binding proteins 3

^a^Self-report

^b^Adjusted for age at diagnosis, education, body mass index (BMI) at baseline, menopausal status at baseline, disease (Charlson comorbidity score 0/≥1), and Tumor, Node, Metastasis (TNM) stage;

^c^Adjusted for study site, age, ethnicity, education, menopausal status, disease stage, adjuvant treatment, tamoxifen use, type II diabetes, and smoking status (the magnitude of differences decreased after for adjusting for BMI too)

#### Hypertension

Nine studies reported findings related to hypertension (Table 6). Three reported adjusted analyses. Bao et al. 2013 reported results that were statistically consistent with parameter values ranging from lower odds to higher odds regarding the association between exercise and hypertension among Chinese women (Referent: No exercise; <3.5 hrs/week, HR:1.01; 95% CI:0.72–1.42; ≥3.5 hrs/week HR:0.94, 95% CI:0.67–1.31) [51]. The comparison groups used by Obi et al. 2014 [52] limited our ability to draw conclusions regarding the magnitude, direction, and precision of the longitudinal association exercise between exercise and hypertension. The third, which included predominantly White women, reported a modest negative longitudinal association between exercise and hypertension, but that data were consistent with parameter values ranging from little to no effect to a strong negative association [47].

**Table 6:** Associations of exercise with hypertension related outcomes

Study	Hypertension Outcome	Findings		
**Adjusted analyses**				
Bao et al. 2013 [51]^b^	Blood pressure (≥ 130/85 mmHg)	Referent: No exercise < 3.5 hrs/week: OR:1.01 (95%CI: 0.72–1.42) ≥ 3.5 hrs/week: OR:0.94 (95%CI: 0.67–1.31)		
Obi et al. 2014 [52]^c^	Hypertension^a+physician−confirmed^	MET-hrs/week		
		0: Referent > 0-<2: HR:0.98 (95% CI: 0.67–1.42) < 24: HR:0.82 (95% CI: 0.56–1.21) < 42: HR:0.95 (95% CI: 0.64–1.40) ≥ 42: HR:0.85 (95% CI: 0.57–1.28)		
Upshaw et al. 2020 [47]^d^	Hypertension^a+clinical chart review^	β = −2.13 (−4.48, 0.21)		
**Unadjusted analyses**				
		**Exercise**		
		**Guidelines**	**Met guidelines**	
			**Yes**	**No**
Ariza-García et al. 2013 [54]	Diastolic blood pressure (mmHg)	Unclear	M (SD)	
			80.9 (9.3)	84.5 (10.0)
	Systolic blood pressure (mmHg)		121.8 (14.8)	125.6 (15.7)
Busen et al. 2023 [46]	High blood pressure^a^	≥ 150 min/week of MPA or ≥ 75 min/week of VPA (ACS/USDHHS)	57.0%	69.2%
Dieli-Conwright et al. 2022 [45]	High blood pressure^a^	≥ 9 MET-hrs/week	39.6%	45.7%
Jones et al. 2016 [43]	Hypertension	≥ 9 MET-hrs/week	35.4%	42.7%
Kim et al. 2021 [48]	Systolic blood pressure (mmHg)	≥ 500 MET-min/week	M (SD)	
			119.8 (14.9)	120.6 (15.3)
Marell et al. 2023 [44]	Hypertension^a^	≥ 150 min/week of MVPA (ACSM, USDHHS, NCCN)	21.5%	40.8%

*HR* Hazards ratio, *MET* Metabolic equivalent, *MPA* moderate-intensity physical activity, *VPA* vigorous-intensity physical activity, *MVPA* moderate-to-vigorous-intensity physical activity, *ACS* American Cancer Society, *USDHHS* U.S. Department of Health and Human Services, *ACSM* American College of Sports Medicine, *NCCN* National Comprehensive Cancer Network

^a^Self-report

^b^Adjusted for age at diagnosis, education, body mass index (BMI) at baseline, menopausal status at baseline, disease (Charlson comorbidity score 0/≥1), and Tumor, Node, Metastasis (TNM) stage

^c^Adjusted for: age (continuous), study region, postmenopausal at breast cancer (BC) diagnosis, marital status, education, BMI at recruitment, age at menarche, menopausal hormone therapy (MHT) at BC diagnosis, parity, smoking habits at BC diagnosis, and alcohol consumption, tumor stage, a combined variable of her2/neu receptor status and trastuzumab medication, and other types of therapy (chemotherapy, radiotherapy, intake of tamoxifen, aromatase inhibitors, trastuzumab, and/or bisphosphonates), and diabetes mellitus at baseline;

^d^Variables adjusted for not reported

Six studies reported cross-sectional unadjusted analyses (Table 6). Four reported that the prevalence of hypertension or high blood pressure was lower among those who met guidelines [43–46]. The two others reported that those who met guidelines had lower systolic [48, 54] and diastolic blood pressure [54].

#### Cholesterol

Seven studies reported findings related to cholesterol (Supplement 6), two of which reported adjusted analyses. Bao et al. 2013 reported results that were statistically consistent with parameter values ranging from lower odds to higher odds regarding the association between exercise and low high-density lipoprotein cholesterol (< 1.3 mmol/l) among Chinese women (Referent: No exercise; <3.5 hrs/week HR:1.10, 95% CI:0.79–1.53; ≥3.5 hrs/week HR:1.01, 95% CI:0.73–1.40) [51]. The other, which included predominantly White women, reported a modest negative longitudinal association between exercise and hyperlipidemia, but parameter values ranged from little to no effect to a strong negative association [47].

Five studies reported cross-sectional unadjusted analyses (Supplement 6). Among those who met guidelines, the prevalence of high cholesterol [45, 46], hyperlipidemia [43] and hypercholesteremia [44] were all lower. A study of Korean women reported lower total cholesterol among those who met guidelines [48].

#### Kidney disease

Marell et al. 2023 [44] compared the unadjusted proportion of those with kidney disease between those who did and did not meet exercise guidelines (≥ 150 min/week) in a cross-sectional study. Among those who met guidelines, none had kidney disease, compared to 5.9% who did not.

#### Comorbidities

Nilsson et al. 2016 [55] compared the unadjusted proportion of those with a comorbidity (hypertension, diabetes, hyperlipidemia) across exercise levels in a cross-sectional study. Forty-five percent of those who were inactive reported a comorbidity, compared to 30% who did some light exercise, and 19% of those who did regular exercise and training/ regular hard physical training for competition sports.

### Muscle-strengthening exercise

Kim et al. 2020 [49] examined the unadjusted association of muscle-strengthening exercise with diabetes among Korean women and found that only 3.1% of those who met muscle-strengthening guidelines had diabetes, compared to 15.9% of those who did not meet guidelines.

## Discussion

Findings stemming from adjusted analyses of longitudinal observational data suggest that aerobic exercise could potentially lower the risk of cardiovascular disease among breast cancer survivors. While there were limited adjusted data for other outcomes of interest, unadjusted analyses of largely cross-sectional data suggest that exercise may be associated with favorable outcomes relating to cardiac function, diabetes, hypertension, and cholesterol and kidney disease. There was limited evidence concerning the association of muscle-strengthening exercise with cardiovascular outcomes [49], and there were no data on variations in the relationship between exercise (aerobic and muscle-strengthening) and cardiovascular health based on race, ethnicity, or socioeconomic characteristics. Our findings extend those of a prior review that evaluated the effect of exercise interventions on cardiovascular health among breast cancer survivors [63]. They also align with previous reviews among the general population, though we were unable to draw conclusions regarding variations in associations [64].

Disparities in cardiovascular health are well documented based on race and/or ethnicity and socioeconomic characteristics [65–68]. However, the studies included in this review provided limited evidence on the variations in the relationship between exercise and cardiovascular outcomes across subgroups defined by race, ethnicity, or socioeconomic status. Apart from studies that focused on Asian [48, 49, 51] or Black [46] women, most studies were comprised of predominantly White women. Further research is needed to quantify variations in the relationship of exercise with cardiovascular health among breast cancer survivors to better understand the reasons underpinning disparities in cardiovascular outcomes.

This review has several limitations. We excluded studies that examined associations broadly among cancer survivors as those studies limited the ability to isolate findings for breast cancer survivors. Studies examining exercise outside the leisure-time domain were excluded, and there is also a possibility of measurement error in relation to exercise due to recall and social desirability bias [69, 70]. We also excluded numerous studies that examined the association between exercise and comorbidities where comorbidities were grouped together and included other cancers, respiratory diseases, osteoarthritis, etc. Studies evaluating the effects of behavioral interventions were excluded as the goal of our review was to evaluate the isolated benefits of “real-world” habitual aerobic and/or muscle strengthening exercise on cardiovascular health.

Prior research indicates that cardiac rehabilitation could help reduce the risk of cardiotoxicity among breast cancer survivors [71, 72]. However, cardiac rehabilitation may involve comprehensive long-term, multidisciplinary interventions encompassing supervised exercise, dietary counseling, and cardiovascular disease risk management [71, 73]. As a result, the effects of cardiac rehabilitation on reducing the risk of cardiotoxicity could be attributable to a combination of benefits offered by exercise, improved diet, and cardiac risk management. Therefore, we excluded studies on cardiac rehabilitation from this review as it would have limited our ability to isolate the effects of exercise alone on cardiovascular health.

The observational studies included in this review had a number of limitations. Most studies predominantly reported cross-sectional analyses, and the quality appraisal revealed that confounders (age, comorbidities, treatments, etc.) were rarely considered in design and analyses. Only seven studies reported adjusted analyses [42, 43, 47, 48, 50–52]. Measurement of exercise (i.e., the exposure) over time may help to establish the temporal association between exercise and cardiovascular health in real-world settings. Many studies relied on self-reported measures from breast cancer survivors, while several studies did not report how data on cardiovascular outcomes were collected. There is a need for objective and/or clinician confirmed measures of cardiovascular outcomes to improve validity and reliability. Additionally, we found only one study that examined the relationship of muscle-strengthening exercise with cardiovascular outcomes. This is consistent with prior reviews that have found fewer studies including muscle-strengthening exercise compared to aerobic exercise [74]. Future research should measure all domains of aerobic physical activity as well as muscle strengthening activity.

There are several ways to strengthen future research examining the relationship between exercise and cardiovascular health. There is a need for more large prospective studies of diverse breast cancer survivors including valid and reliable measures of exercise and cardiovascular health outcomes to evaluate possible variations across race and/or ethnicity, socioeconomic status, and geographic location. There is a need for purposeful collection of data from underrepresented and underserved women to address these research gaps. Future longitudinal studies may facilitate evaluations of the relationship between exercise and cardiovascular health. Researchers should also consider using consistent measurements of survivor characteristics, exercise, and cardiovascular health outcomes to facilitate cross-study comparisons and the pooling of data across studies.

In summary, the most recent exercise guidelines concluded that the impact of exercise to prevent or improve cardiotoxicity is an emerging field, and that more research is needed to understand the impact of exercise on cardiac and vascular function [18]. Our review suggests that aerobic exercise offers breast cancer survivors some protection against adverse cardiovascular health outcomes. Findings of this review also align with exercise providing favorable changes relating to adiposity, inflammation, immune function, metabolic regulation, and sex hormones [75]. Much more research is needed to more precisely establish the dose-response relationship of aerobic and muscle-strengthening exercise with cardiovascular health among breast cancer survivors, as well as the underpinning physiological mechanisms. Importantly, it is currently unclear whether the relationship of aerobic or muscle-strengthening exercise with cardiovascular health differs based on race, ethnicity, or socioeconomic status. Future researchers should consider recruiting more diverse samples of breast cancer survivors and/or pooling data to be able to make these comparisons.

## Supplementary Information

Supplementary Material 1. [76–90]

## Data Availability

No datasets were generated or analysed during the current study.

## References

[1] Galimzhanov A, Istanbuly S, Tun HN, Ozbay B, Alasnag M, Ky B, et al. Cardiovascular outcomes in breast cancer survivors: a systematic review and meta-analysis. EJPC. 2023;30(18):2018–31. 10.1093/eurjpc/zwad243 37499186

[2] Mehta LS, Watson KE, Barac A, Beckie TM, Bittner V, Cruz-Flores S, et al. Cardiovascular disease and breast cancer: where these entities intersect: a scientific statement from the American Heart Association. Circulation. 2018;137(8):e30-66. 29437116 10.1161/CIR.0000000000000556 PMC6722327

[3] National Cancer Institute. Cardiotoxicity. 2024. Available from: https://prevention.cancer.gov/major-programs/supportive-care-and-symptom-management/cardiotoxicity.

[4] Bostany G, Chen Y, Francisco L, Dai C, Meng Q, Sparks J, et al. Cardiac dysfunction among breast cancer survivors: role of cardiotoxic therapy and cardiovascular risk factors. J Clin Oncol. 0(0):JCO.23.01779. 10.1200/JCO.23.01779 38833638

[5] Kwan ML, Sternfeld B, Ergas IJ, Timperi AW, Roh JM, Hong C-C, et al. Change in physical activity during active treatment in a prospective study of breast cancer survivors. Breast Cancer Res Treat. 2012;131(2):679–90. 21953007 10.1007/s10549-011-1788-4 PMC3273453

[6] Brunet J, Taran S, Burke S, Sabiston CM. A qualitative exploration of barriers and motivators to physical activity participation in women treated for breast cancer. Disabil Rehabil. 2013;35(24):2038–45. 23772995 10.3109/09638288.2013.802378

[7] Thompson CL, Owusu C, Nock NL, Li L, Berger NA. Race, age, and obesity disparities in adult physical activity levels in breast cancer patients and controls. Front Public Health. 2014;2:150. 25285306 10.3389/fpubh.2014.00150 PMC4168674

[8] Irwin ML, Crumley D, McTiernan A, Bernstein L, Baumgartner R, Gilliland FD, et al. Physical activity levels before and after a diagnosis of breast carcinoma: the Health, Eating, Activity, and Lifestyle (HEAL) study. Cancer. 2003;97(7):1746–57. 12655532 10.1002/cncr.11227 PMC3034406

[9] Irwin ML, McTiernan A, Bernstein L, Gilliland FD, Baumgartner R, Baumgartner K, Ballard-Barbash R. Physical activity levels among breast cancer survivors. Med Sci Sport Exerc. 2004;36(9):1484. PMC3000611 15354027

[10] Bull FC, Al-Ansari SS, Biddle S, Borodulin K, Buman MP, Cardon G, et al. World Health Organization 2020 guidelines on physical activity and sedentary behaviour. Br J Sports Med. 2020;54(24):1451. 33239350 10.1136/bjsports-2020-102955 PMC7719906

[11] Kraus WE, Powell KE, Haskell WL, Janz KF, Campbell WW, Jakicic JM, et al. Physical activity, all-cause and cardiovascular mortality, and cardiovascular disease. Med Sci Sports Exerc. 2019;51(6):1270–81. 31095084 10.1249/MSS.0000000000001939 PMC6527136

[12] Isath A, Koziol KJ, Martinez MW, Garber CE, Martinez MN, Emery MS, et al. Exercise and cardiovascular health: a state-of-the-art review. Prog Cardiovasc Dis. 2023;79:44–52. 37120119 10.1016/j.pcad.2023.04.008

[13] Wojcik KM, Wilson OWA, Sheils MS, Sheppard VL, Jayasekera J. Racithnic, and socioeconomic disparities in meeting physical activity guidelines among female breast cancer survivors in the United States. Cancer Epidemiol Biomarkers Prev. 2024;33:1610. 39269270 10.1158/1055-9965.EPI-24-0650 PMC11609821

[14] Olson EA, Mullen SP, Rogers LQ, Courneya KS, Verhulst S, McAuley E. Meeting physical activity guidelines in rural breast cancer survivors. Am J Health Behav. 2014;38(6):890–9. 25341266 10.5993/ajhb.38.6.11 PMC4209408

[15] Rogers LQ, Markwell SJ, Courneya KS, McAuley E, Verhulst S. Physical activity type and intensity among rural breast cancer survivors: patterns and associations with fatigue and depressive symptoms. J Cancer Surviv. 2011;5(1):54–61. 21110134 10.1007/s11764-010-0160-8 PMC3041842

[16] Sutton AL, Felix AS, Wahl S, Franco RL, Leicht Z, Williams KP, et al. Racial disparities in treatment-related cardiovascular toxicities amongst women with breast cancer: a scoping review. J Cancer Surviv. 2023;17(6):1596–605. 35420375 10.1007/s11764-022-01210-2

[17] Vo JB, Ramin C, Lawrence WR, Barac A, Ho KL, Rhee J, et al. Racial and ethnic disparities in treatment-related heart disease mortality among US breast cancer survivors. JNCI Cancer Spectr. 2023;7(2):pkad024. 36943362 10.1093/jncics/pkad024 PMC10130190

[18] Campbell KL, Winters-Stone KM, Wiskemann J, May AM, Schwartz AL, Courneya KS, et al. Exercise guidelines for cancer survivors: consensus statement from international multidisciplinary roundtable. Med Sci Sports Exerc. 2019;51(11):2375–90. 31626055 10.1249/MSS.0000000000002116 PMC8576825

[19] Physical Activity Guidelines Advisory Committee. 2018 Physical activity guidelines advisory committee scientific report. Washington, DC: USDHHS; 2018.

[20] Ligibel JA, Bohlke K, May AM, Clinton SK, Demark-Wahnefried W, Gilchrist SC, et al. Exercise, diet, and weight management during cancer treatment: ASCO guideline. J Clin Oncol. 2022;40(22):2491–507. 35576506 10.1200/JCO.22.00687

[21] Lee K, Tripathy D, Demark-Wahnefried W, Courneya KS, Sami N, Bernstein L, et al. Effect of aerobic and resistance exercise intervention on cardiovascular disease risk in women with early-stage breast cancer: a randomized clinical trial. JAMA Oncol. 2019;5(5):710–4. 30920602 10.1001/jamaoncol.2019.0038 PMC6512455

[22] Kong L, Gao R. Aerobic exercise combined with resistance exercise training improves cardiopulmonary function and blood lipid of patients with breast cancer: a systematic review and meta-analysis. Medicine (Baltimore). 2022;101(51);e32391. 10.1097/MD.0000000000032391 PMC9794326 36595800

[23] Al-Mhanna SB, Batrakoulis A, Norhayati MN, Mohamed M, Drenowatz C, Irekeola AA, et al. Combined aerobic and resistance training improves body composition, alters cardiometabolic risk, and ameliorates cancer-related indicators in breast cancer patients and survivors with overweight/obesity: a systematic review and meta-analysis of randomized controlled trials. J Sports Sci Med. 2024;23(2):366–95. 38841642 10.52082/jssm.2024.366 PMC11149074

[24] Courneya KS. Efficacy, effectiveness, and behavior change trials in exercise research. Int J Behav Nutr Phys Act. 2010;7(1):81. 21073717 10.1186/1479-5868-7-81 PMC2989301

[25] Hirko KA, Rocque G, Reasor E, Taye A, Daly A, Cutress RI, et al. The impact of race and ethnicity in breast cancer-disparities and implications for precision oncology. BMC Med. 2022;20(1):72. 35151316 10.1186/s12916-022-02260-0 PMC8841090

[26] Oyer RA, Hurley P, Boehmer L, Bruinooge SS, Levit K, Barrett N, et al. Increasing racial and ethnic diversity in cancer clinical trials: an American Society of Clinical Oncology and Association of Community Cancer Centers joint research statement. J Clin Oncol. 2022;40(19):2163–71. 35588469 10.1200/JCO.22.00754

[27] Arksey H, O’Malley L. Scoping studies: towards a methodological framework. Int J Soc Res Methodol. 2005;8(1):19–32.

[28] Levac D, Colquhoun H, O’Brien KK. Scoping studies: advancing the methodology. Implement Sci. 2010;5(1):69. 20854677 10.1186/1748-5908-5-69 PMC2954944

[29] Peters M, Godfrey C, McInerney P, Munn Z, Tricco A, Khalil H. Chapter 11: scoping reviews (2020 version). In: JBI manual for evidence synthesis. 2020. https://synthesismanual.jbi.global. 10.11124/JBIES-20-00167 33038124

[30] Tricco AC, Lillie E, Zarin W, O’Brien KK, Colquhoun H, Levac D, et al. PRISMA extension for scoping reviews (PRISMA-ScR): checklist and explanation. Ann Intern Med. 2018;169(7):467–73. 30178033 10.7326/M18-0850

[31] American Cancer Society. Survivorship: during and after treatment. 2024. Available from: https://www.cancer.org/cancer/survivorship.html.

[32] Quinn TD, Barone Gibbs B. Context matters: the importance of physical activity domains for public health. J Meas Phys Behav. 2023;6(4):245–9.

[33] American Heart Association. What is cardiovascular disease? 2024.

[34] Centers for Disease Control and Prevention. Know your risk for heart disease. 2024. Available from: https://www.cdc.gov/heart-disease/risk-factors/.

[35] Veritas Health Innovation. Covidence systematic review software. Melbourne; 2023. Available from: https://www.covidence.org.

[36] Hong QN, Pluye P, Fàbregues S, Bartlett G, Boardman F, Cargo M, et al. Mixed methods appraisal tool (MMAT). Canada: Canadian Intellectual Property Office; 2018.

[37] Zaza S, Wright-De Agüero LK, Briss PA, Truman BI, Hopkins DP. Data collection instrument and procedure for systematic reviews in the guide to community preventive services. Am J Prev Med. 2000;188(Suppl 1):44–74. 10.1016/s0749-3797(99)00122-1 10806979

[38] McKenzie JE, Brennan SE. Chapter 12: Synthesizing and presenting findings using other methods. In: Higgins J, Thomas J, Chandler J, Cumpston M, Li T, Page M, Welch V, editors. Cochrane handbook for systematic reviews of interventions. Version 6.42024.

[39] Farland LV, Correia KF, Wise LA, Williams PL, Ginsburg ES, Missmer SA. P-values and reproductive health: what can clinical researchers learn from the American Statistical Association? Hum Reprod. 2016;31(11):2406–10. 27664212 10.1093/humrep/dew192 PMC5088632

[40] Rothman KJ. Huybrechtsm KF, Murray EJ. Chapter 8: Random error and the role of statistics. In: Rothman KJ, editor. Epidemiology: an introduction. 2nd ed. Oxford: Oxford Univ Press; 2012. p. 148–63.

[41] Savitz DA, Wise LA, Bond JC, Hatch EE, Ncube CN, Wesselink AK, et al. Responding to reviewers and editors about statistical significance testing. Ann Intern Med. 2024;177(3):385–6. 38373303 10.7326/M23-2430

[42] Irwin ML, McTiernan A, Bernstein L, Gilliland FD, Baumgartner R, Baumgartner K, Ballard-Barbash R. Relationship of obesity and physical activity with C-peptide, leptin, and insulin-like growth factors in breast cancer survivors. Cancer Epidemiol Biomarkers Prev. 2005;14(12):2881–8. 16365005 10.1158/1055-9965.EPI-05-0185 PMC3000615

[43] Jones LW, Habel LA, Weltzien E, Castillo A, Gupta D, Kroenke CH, et al. Exercise and risk of cardiovascular events in women with nonmetastatic breast cancer. J Clin Oncol. 2016;34(23):2743–9. 27217451 10.1200/JCO.2015.65.6603 PMC5019746

[44] Marell PS, Vierkant RA, Olson JE, Herrmann J, Larson NL, Lebrasseur NK, et al. Changes in amount and intensity of physical activity over time in breast cancer survivors. JNCI Cancer Spectr. 2023;7(5);pkad056. 37561108 10.1093/jncics/pkad056 PMC10471529

[45] Dieli-Conwright CM, Nelson RA, Simon MS, Irwin ML, Neuhouser ML, Reding KW, et al. Cardiometabolic risk factors, physical activity, and postmenopausal breast cancer mortality: results from the women’s health initiative. BMC Womens Health. 2022;22(1):32. 35120497 10.1186/s12905-022-01614-3 PMC8817588

[46] Busen K, Sanderson M, Banks AD, Wallace H, Nechuta S. Patterns of physical activity and the role of obesity and comorbidities among long-term African American breast cancer survivors. J Racial Ethn Health Disparities. 2023;10(5):2261–72. 36071314 10.1007/s40615-022-01405-4 PMC10170401

[47] Upshaw JN, Hubbard RA, Hu J, Brown JC, Smith AM, Demissei B, et al. Physical activity during and after breast cancer therapy and associations of baseline physical activity with changes in cardiac function by echocardiography. Cancer Med. 2020;9(17):6122–31. 32645252 10.1002/cam4.3277 PMC7476829

[48] Kim KH, Choi S, Kim K, Chang J, Kim SM, Kim SR, et al. Association between physical activity and subsequent cardiovascular disease among 5-year breast cancer survivors. Breast Cancer Res Treat. 2021;188(1):203–14. 33599866 10.1007/s10549-021-06140-8

[49] Kim M, So WY, Kim J. Relationships between exercise modality and activity restriction, quality of life, and hematopoietic profile in Korean breast cancer survivors. Int J Env Res Pub He. 2020;17:18. 10.3390/ijerph17186899 PMC7559845 32967252

[50] Peck SS, Esmaeilzadeh M, Rankin K, Shalmon T, Fan CS, Somerset E, et al. Self-reported physical activity, QoL, cardiac function, and cardiorespiratory fitness in women with HER2 + breast cancer. JACC CardioOncol. 2022;4(3):387–400. 36213351 10.1016/j.jaccao.2022.06.006 PMC9537092

[51] Bao PP, Zheng Y, Nechuta S, Gu K, Cai H, Peng P, et al. Exercise after diagnosis and metabolic syndrome among breast cancer survivors: a report from the Shanghai breast cancer survival study. Cancer Causes Control. 2013;24(9):1747–56. 23860950 10.1007/s10552-013-0252-7 PMC3858324

[52] Obi N, Gornyk D, Heinz J, Vrieling A, Seibold P, Chang-Claude J, Flesch-Janys D. Determinants of newly diagnosed comorbidities among breast cancer survivors. J Cancer Surviv. 2014;8(3):384–93. 24570215 10.1007/s11764-013-0338-y

[53] Guinan EM, Connolly EM, Kennedy MJ, Hussey J. The presentation of metabolic dysfunction and the relationship with energy output in breast cancer survivors: a cross-sectional study. Nutr J. 2013;12: 99. 23855321 10.1186/1475-2891-12-99 PMC3717288

[54] Ariza-García A, Galiano-Castillo N, Cantarero-Villanueva I, Fernández-Lao C, Díaz-Rodríguez L, Arroyo-Morales M. Influence of physical inactivity in psychophysiological state of breast cancer survivors. Eur J Cancer Care. 2013;22(6):738–45. 10.1111/ecc.12101 23889104

[55] Nilsson H, Angerås U, Bock D, Börjesson M, Onerup A, Fagevik Olsen M, et al. Is preoperative physical activity related to post-surgery recovery? A cohort study of patients with breast cancer. BMJ Open. 2016;6(1):e007997. 26769776 10.1136/bmjopen-2015-007997 PMC4735182

[56] Kriska A. Modifiable activity questionnaire. Med Sci Sports Exerc. 1997;29(Suppl):S73–8.

[57] Garber CE, Blissmer B, Deschenes MR, Franklin BA, Lamonte MJ, Lee IM, et al. Quantity and quality of exercise for developing and maintaining cardiorespiratory, musculoskeletal, and neuromotor fitness in apparently healthy adults: Guidance for prescribing exercise. Med Sci Sports Exerc. 2011;43(7):1334–59. 21694556 10.1249/MSS.0b013e318213fefb

[58] Rock CL, Thomson CA, Sullivan KR, Howe CL, Kushi LH, Caan BJ, et al. American Cancer Society nutrition and physical activity guideline for cancer survivors. CA Cancer J Clin. 2022;72(3):230–62. 35294043 10.3322/caac.21719

[59] Sanft T, Day A, Peterson L, Rodriguez MA, Ansbaugh S, Armenian S, et al. NCCN guidelines^®^ insights: survivorship, version 1.2022: featured updates to the NCCN guidelines. JNCCN. 2022;20(10):1080–90. 36240847 10.6004/jnccn.2022.0052

[60] Irwin ML, Duggan C, Wang CY, Smith AW, McTiernan A, Baumgartner RN, et al. Fasting C-peptide levels and death resulting from all causes and breast cancer: the health, eating, activity, and lifestyle study. J Clin Oncol. 2011;29(1):47–53. 21115859 10.1200/JCO.2010.28.4752 PMC3055859

[61] Wayne S, Neuhouser ML, Ulrich CM, Koprowski C, Wiggins C, Baumgartner KB, et al. Association between alcohol intake and serum sex hormones and peptides differs by tamoxifen use in breast cancer survivors. Cancer Epidemiol Biomarkers Prev. 2008;17(11):3224–32. 18957523 10.1158/1055-9965.EPI-08-0171 PMC2673729

[62] Wayne SJ, Neuhouser ML, Ulrich CM, Koprowski C, Baumgartner KB, Baumgartner RN, et al. Dietary fiber is associated with serum sex hormones and insulin-related peptides in postmenopausal breast cancer survivors. Breast Cancer Res Treat. 2008;112(1):149–58. 18058020 10.1007/s10549-007-9834-y

[63] Lee K. Clinical exercise prescription for cardiovascular health in breast cancer survivors. KJSM. 2021;39(3):77–90.

[64] Shiroma EJ, Lee IM. Physical activity and cardiovascular health. Circ. 2010;122(7):743–52. 10.1161/CIRCULATIONAHA.109.914721 20713909

[65] Javed Z, Haisum Maqsood M, Yahya T, Amin Z, Acquah I, Valero-Elizondo J, et al. Race, racism, and cardiovascular health: applying a social determinants of health framework to racial/ethnic disparities in cardiovascular disease. Circulation. 2022;15(1):e007917. 35041484 10.1161/CIRCOUTCOMES.121.007917

[66] Jerome GJ, Boyer WR, Bustamante EE, Kariuki J, Lopez-Jimenez F, Paluch AE, et al. Increasing equity of physical activity promotion for optimal cardiovascular health in adults: a scientific statement from the American Heart Association. Circulation. 2023;147(25):1951–62. 37222169 10.1161/CIR.0000000000001148

[67] Mehta LS, Velarde GP, Lewey J, Sharma G, Bond RM, Navas-Acien A, et al. Cardiovascular disease risk factors in women: the impact of race and ethnicity: a scientific statement from the American Heart Association. Circulation. 2023;147(19):1471–87. 37035919 10.1161/CIR.0000000000001139 PMC11196122

[68] Schultz WM, Kelli HM, Lisko JC, Varghese T, Shen J, Sandesara P, et al. Socioeconomic status and cardiovascular outcomes: challenges and interventions. Circulation. 2018;137(20):2166–78. 29760227 10.1161/CIRCULATIONAHA.117.029652 PMC5958918

[69] Adams SA, Matthews CE, Ebbeling CB, Moore CG, Cunningham JE, Fulton J, Hebert JR. The effect of social desirability and social approval on self-reports of physical activity. Am J Epidemiol. 2005;161(4):389–98. 15692083 10.1093/aje/kwi054 PMC2958515

[70] Brenner PS, DeLamater JD. Social desirability bias in self-reports of physical activity: is an exercise identity the culprit? Soc Indic Res. 2014;117(2):489–504.

[71] Franklin BA, Brinks J. Cardiac rehabilitation: underrecognized/underutilized. Curr Treat Options Cardiovasc Med. 2015;17(12):62. 26526338 10.1007/s11936-015-0422-x

[72] Chang A, Boyd A, Leung I, Trejo E, Dixit N, Mallidi J, et al. Formative research to adapt a cardiac rehabilitation program to breast cancer survivors: the heart health after cancer treatment (HEART-ACT) study. Cardio-Oncology. 2024;10(1):28. 38760873 10.1186/s40959-024-00228-y PMC11100255

[73] Kirkham Amy A, Mackey John R, Thompson Richard B, Haykowsky Mark J, Oudit Gavin Y, McNeely M, et al. TITAN trial. JACC Adv. 2023;2(6):100424. 38939428 10.1016/j.jacadv.2023.100424 PMC11198667

[74] Wilson OWA, Wojcik KM, Kamil D, Gorzelitz J, Butera G, Matthews CE, Jayasekera J. The associations of muscle-strengthening exercise with recurrence and mortality among breast cancer survivors: a systematic review. Int J Behav Nutr Phys Act. 2024;21(1):100. 39256770 10.1186/s12966-024-01644-0 PMC11389293

[75] McTiernan A. Mechanisms linking physical activity with cancer. Nat Rev Cancer. 2008;8(3):205–11. 18235448 10.1038/nrc2325

[76] National Breast Cancer Foundation. Stages. 2022. Available from: https://www.nationalbreastcancer.org/breast-cancer-staging/.

[77] American College of Sports Medicine. ACSM’s guidelines for exercising testing and prescription. 10th ed. Indianapolis: American College of Sports Medicine; 2018.

[78] Godin G. The Godin-Shephard leisure-time physical activity questionnaire. HFJC. 2011;4(1):18–22.

[79] Matthews CE, Shu X-O, Yang G, Jin F, Ainsworth BE, Liu D, et al. Reproducibility and validity of the Shanghai women’s health study physical activity questionnaire. Am J Epidemiol. 2003;158(11):1114–22. 14630608 10.1093/aje/kwg255

[80] Grundy SM, Cleeman JI, Daniels SR, Donato KA, Eckel RH, Franklin BA, et al. Diagnosis and management of the metabolic syndrome: an American Heart Association/National Heart, Lung, and Blood Institute scientific statement. Circulation. 2005;112:2735–52. 16157765 10.1161/CIRCULATIONAHA.105.169404

[81] Schmidt ME, Chang-Claude J, Vrieling A, Seibold P, Heinz J, Obi N, et al. Association of pre-diagnosis physical activity with recurrence and mortality among women with breast cancer. Int J Cancer. 2013;133(6):1431–40. 23444048 10.1002/ijc.28130

[82] Richardson MT, Leon AS, Jacobs DR Jr, Ainsworth BE, Serfass R. Comprehensive evaluation of the Minnesota leisure time physical activity questionnaire. J Clin Epidemiol. 1994;47(3):271–81. 8138837 10.1016/0895-4356(94)90008-6

[83] Craig CL, Marshall AL, Sjöström M, Bauman AE, Booth ML, Ainsworth BE, et al. International physical activity questionnaire: 12-country reliability and validity. Med Sci Sports Exerc. 2003;35(8):1381–95. 12900694 10.1249/01.MSS.0000078924.61453.FB

[84] Elosua R, Garcia M, Aguilar A, Molina L, Covas MI, Marrugat J. Validation of the Minnesota leisure time physical activity questionnaire in Spanish women. Investigators of the MARATDON group. Med Sci Sports Exerc. 2000;32(8):1431–7. 10949009 10.1097/00005768-200008000-00011

[85] Amireault S, Godin G, Lacombe J, Sabiston CM. Validation of the Godin-Shephard leisure-time physical activity questionnaire classification coding system using accelerometer assessment among breast cancer survivors. J Cancer Surviv. 2015;9(3):532–40. 25666749 10.1007/s11764-015-0430-6

[86] Sternfeld B, Weltzien E, Quesenberry CP Jr, Castillo AL, Kwan M, Slattery ML, Caan BJ. Physical activity and risk of recurrence and mortality in breast cancer survivors: findings from the LACE study. Cancer Epidemiol Biomarkers Prev. 2009;18(1):87–95. 19124485 10.1158/1055-9965.EPI-08-0595 PMC3507507

[87] Staten LK, Taren DL, Howell WH, Tobar M, Poehlman ET, Hill A, et al. Validation of the Arizona activity frequency questionnaire using doubly labeled water. Med Sci Sports Exerc. 2001;33(11):1959–67. 11689750 10.1097/00005768-200111000-00024

[88] Groarke JD, Cheng S, Moslehi J. Cancer-drug discovery and cardiovascular surveillance. N Engl J Med. 2013;369(19):1779–81. 24180496 10.1056/NEJMp1313140

[89] Godin G, Shephard RJ. A simple method to assess exercise behavior in the community. Can J Appl Sci. 1985;10:141–6. 4053261

[90] Anderson GL, Manson J, Wallace R, Lund B, Hall D, Davis S, et al. Implementation of the women’s health initiative study design. Ann Epidemiol. 2003;13(9):S5-17. 14575938 10.1016/s1047-2797(03)00043-7

