# Supplementary material for: Exercise and cardiovascular health among breast cancer survivors: a scoping review of current observational evidence
Source: Cardiooncology. 2025 Feb 26;11:24. doi: 10.1186/s40959-025-00310-z (PMC11863919; doi:10.1186/s40959-025-00310-z)
Supplement: Supplementary file 1 — Supplementary Material 1. [76–90] [file 40959_2025_310_MOESM1_ESM.docx]

**Supplement 1. PRISMA-ScR Checklist**

| **SECTION** | **ITEM** | **PRISMA-ScR CHECKLIST ITEM** | **REPORTED ON PAGE #** |
| --- | --- | --- | --- |
| **TITLE** | | | |
| Title | 1 | Identify the report as a scoping review. | 1 |
| **ABSTRACT** | | | |
| Structured summary | 2 | Provide a structured summary that includes (as applicable): background, objectives, eligibility criteria, sources of evidence, charting methods, results, and conclusions that relate to the review questions and objectives. | 2 |
| **INTRODUCTION** | | | |
| Rationale | 3 | Describe the rationale for the review in the context of what is already known. Explain why the review questions/objectives lend themselves to a scoping review approach. | 3-4 |
| Objectives | 4 | Provide an explicit statement of the questions and objectives being addressed with reference to their key elements (e.g., population or participants, concepts, and context) or other relevant key elements used to conceptualize the review questions and/or objectives. | 4 |
| **METHODS** | | | |
| Protocol and registration | 5 | Indicate whether a review protocol exists; state if and where it can be accessed (e.g., a Web address); and if available, provide registration information, including the registration number. | 5 |
| Eligibility criteria | 6 | Specify characteristics of the sources of evidence used as eligibility criteria (e.g., years considered, language, and publication status), and provide a rationale. | 6, 43 |
| Information sources* | 7 | Describe all information sources in the search (e.g., databases with dates of coverage and contact with authors to identify additional sources), as well as the date the most recent search was executed. | 6 |
| Search | 8 | Present the full electronic search strategy for at least 1 database, including any limits used, such that it could be repeated. | 5, 35-41 |
| Selection of sources of evidence† | 9 | State the process for selecting sources of evidence (i.e., screening and eligibility) included in the scoping revie | 6-7 |
| Data charting process‡ | 10 | Describe the methods of charting data from the included sources of evidence (e.g., calibrated forms or forms that have been tested by the team before their use, and whether data charting was done independently or in duplicate) and any processes for obtaining and confirming data from investigators. | 7 |
| Data items | 11 | List and define all variables for which data were sought and any assumptions and simplifications made. | 7 |
| Critical appraisal of individual sources of evidence§ | 12 | If done, provide a rationale for conducting a critical appraisal of included sources of evidence; describe the methods used and how this information was used in any data synthesis (if appropriate) | 7 |
| Synthesis of results | 13 | Describe the methods of handling and summarizing the data that were charted. | 7-8 |
| **RESULTS** | | | |
| Selection of sources of evidence | 14 | Give numbers of sources of evidence screened, assessed for eligibility, and included in the review, with reasons for exclusions at each stage, ideally using a flow diagram. | 8, 44 |
| Characteristics of sources of evidence | 15 | For each source of evidence, present characteristics for which data were charted and provide the citations. | 8-12 |
| Critical appraisal within sources of evidence | 16 | If done, present data on critical appraisal of included sources of evidence (see item 12). | 12, 45 |
| Results of individual sources of evidence | 17 | For each included source of evidence, present the relevant data that were charted that relate to the review questions and objectives. | 12-22, 46-47 |
| Synthesis of results | 18 | Summarize and/or present the charting results as they relate to the review questions and objectives. | 12-22, 46-47 |
| **DISCUSSION** | | | |
| Summary of evidence | 19 | Summarize the main results (including an overview of concepts, themes, and types of evidence available), link to the review questions and objectives, and consider the relevance to key groups. | 22-23 |
| Limitations | 20 | Discuss the limitations of the scoping review process. | 23-24 |
| Conclusions | 21 | Provide a general interpretation of the results with respect to the review questions and objectives, as well as potential implications and/or next steps. | 24-25 |
| **FUNDING** | | | |
| Funding | 22 | Describe sources of funding for the included sources of evidence, as well as sources of funding for the scoping review. Describe the role of the funders of the scoping review. | 25-26 |

JBI = Joanna Briggs Institute; PRISMA-ScR = Preferred Reporting Items for Systematic reviews and Meta-Analyses extension for Scoping Reviews.

* Where *sources of evidence* (see second footnote) are compiled from, such as bibliographic databases, social media platforms, and Web sites.

† A more inclusive/heterogeneous term used to account for the different types of evidence or data sources (e.g., quantitative and/or qualitative research, expert opinion, and policy documents) that may be eligible in a scoping review as opposed to only studies. This is not to be confused with *information sources* (see first footnote).

‡ The frameworks by Arksey and O’Malley and Levac and colleagues and the JBI guidance refer to the process of data extraction in a scoping review as data charting*.*

§ The process of systematically examining research evidence to assess its validity, results, and relevance before using it to inform a decision. This term is used for items 12 and 19 instead of "risk of bias" (which is more applicable to systematic reviews of interventions) to include and acknowledge the various sources of evidence that may be used in a scoping review (e.g., quantitative and/or qualitative research, expert opinion, and policy document).

**Supplement 2. Search strategy development and strategies**

Database: PubMed/MEDLINE
Platform: National Library of Medicine
Date Searched: 10/04/2024

Date Limits: 1/1/2003 – 10/04/2024

|  | Concept: | Search Strategy: |
| --- | --- | --- |
| #1 | Breast Cancer | "Breast Neoplasms"[Mesh] OR (("Breast"[Mesh] OR "breast*"[Title/Abstract]) AND ("cancer*"[Title/Abstract] OR "neoplas*"[Title/Abstract] OR "carcinoma*"[Title/Abstract] OR "tumor*"[Title/Abstract] OR "tumour*"[Title/Abstract] OR "malignan*"[Title/Abstract] OR "sarcoma*"[Title/Abstract] OR "lymphoma*"[Title/Abstract])) |
| #2 | Physical Activity | "Exercise"[Mesh] OR "Exercise Movement Techniques"[Mesh] OR "Exercise Therapy"[Mesh] OR "exercise*"[Title/Abstract] OR "Physical Fitness"[Mesh] OR "physical fitness"[Title/Abstract] OR "physical activit*"[Title/Abstract] OR "physical performanc*"[Title/Abstract] OR "Physical Exertion"[Mesh] OR "physical exertion"[Title/Abstract] OR "Physical Endurance"[Mesh] OR "physical endurance"[Title/Abstract:~4] OR "endurance training"[Title/Abstract] OR "physical intensit*"[Title/Abstract] OR "Recreation"[Mesh:NoExp] OR "recreational activit*"[Title/Abstract] OR "Sports"[Mesh] OR "sport*"[Title/Abstract] OR "stretching"[Title/Abstract] OR "Yoga"[Mesh] OR "yoga"[Title/Abstract] OR "Tai Ji"[Mesh] OR "Tai Ji"[Title/Abstract] OR "Tai Chi"[Title/Abstract] OR "muscle strength*"[Title/Abstract] OR "muscle strengthening"[Title/Abstract:~4] OR "resistance training"[Title/Abstract] OR "strength training"[Title/Abstract:~4] OR "muscle strength*"[Title/Abstract] OR "weight training"[Title/Abstract] OR "weightlifting"[Title/Abstract] OR "weight lifting"[Title/Abstract] OR "Walking"[Mesh] OR "walk*"[Title/Abstract] OR "gait"[Title/Abstract] OR "jog"[Title/Abstract] OR "jogging"[Title/Abstract] OR "running"[Title/Abstract] OR "swimming"[Title/Abstract] OR "aerobic*"[Title/Abstract] OR "exertion*"[Title/Abstract] OR "isometric*"[Title/Abstract] OR "energy expenditure*"[Title/Abstract] OR "energy balanc*"[Title/Abstract] OR "energy intake*"[Title/Abstract] OR "circuit*"[Title/Abstract] OR "high intensity interval*"[Title/Abstract] OR "high intensity intermittent*"[Title/Abstract] OR "HIIT"[Title/Abstract] OR "sprint interval*"[Title/Abstract] OR "stair climbing"[Title/Abstract:~3] OR "calisthenic*"[Title/Abstract] OR "cycling*"[Title/Abstract] OR "biking*"[Title/Abstract] OR "Sedentary Behavior"[Mesh] OR "sedentary"[Title/Abstract] OR "physical inactivit*"[Title/Abstract] |
| #3 | Mortality & Recurrence | "Neoplasm Recurrence, Local"[Mesh] OR "Recurrence"[Mesh] OR "recurren*"[Title/Abstract] OR "reoccurren*"[Title/Abstract] OR "relaps*"[Title/Abstract] OR "Neoplasms, Second Primary"[Mesh] OR "secondary events"[Title/Abstract] OR "second cancer*"[Title/Abstract] OR "second primary cancer*"[Title/Abstract] OR "secondary cancer*"[Title/Abstract] OR "secondary primary cancer*"[Title/Abstract] OR "Disease Progression"[Mesh] OR "progression*"[Title/Abstract] OR "Disease-Free Survival"[Mesh] OR "cancer survivor*"[Title/Abstract] OR "cancer survival"[Title/Abstract:~3] OR "cause death"[Title/Abstract:~3] OR "all cause"[Title/Abstract] OR "fatal*"[Title/Abstract] OR "Mortality"[Mesh] OR "mortality" [Subheading] OR "mortalit*"[Title/Abstract] OR "Quality of Life"[Mesh] OR "quality of life"[Title/Abstract] OR "life quality"[Title/Abstract] OR "HRQOL"[Title/Abstract] |
| #4 | Limits & Filters | ((#1 AND #2 AND #3) NOT ("Animals"[Mesh] NOT ("Animals"[Mesh] AND "Humans"[Mesh]))) NOT (editorial[Publication Type] OR comment[Publication Type] OR "commentary*"[Title/Abstract] OR news[Publication Type] OR letter[Publication Type] OR retracted publication[Publication Type] OR retraction of publication[Publication Type] OR "retraction of publication*"[Title/Abstract] OR "retraction notice"[Title] OR "retracted publication"[Title] OR "Congress"[Publication Type] OR "Consensus Development Conference"[Publication Type] OR "conference abstract*"[Title/Abstract] OR "conference proceeding*"[Title/Abstract] OR "conference paper*"[Title/Abstract] OR "conference review*"[Title/Abstract] OR "symposium*"[Title/Abstract] OR "Case Reports" [Publication Type] OR "case report*"[Title/Abstract] OR "Review" [Publication Type] OR "review"[Title] OR "Systematic Review" [Publication Type] OR "Systematic Reviews as Topic"[Mesh] OR "systematic review"[Title/Abstract] OR "Meta-Analysis" [Publication Type] OR "Meta-Analysis as Topic"[Mesh] OR "meta-analysis "[Title/Abstract] OR "meta-analyses "[Title/Abstract] OR "protocol"[Title] OR "protocols"[Title] OR "Practice Guideline"[Publication Type] OR "guideline*"[Title]) Filters: English, from 2003/1/1 - 2023/9/18 |

Database: Cochrane CENTRAL
Platform: Wiley & Sones
Date Searched: 10/04/2024

Date Limits: 1/1/2003 – 10/04/2024

|  | Concept: | Search Strategy: |
| --- | --- | --- |
| #1 | Breast Cancer | ([mh "Breast Neoplasms"] OR (([mh "Breast"] OR breast*) NEAR/4 (cancer* OR neoplas* OR carcinoma* OR tumor* OR tumour* OR malignan* OR sarcoma* OR lymphoma*))):ti,ab,kw |
| #2 | Physical Activity | ([mh "Exercise"] OR [mh "Exercise Movement Techniques"] OR [mh "Exercise Therapy"] OR exercise* OR [mh "Physical Fitness"] OR "physical fitness" OR (physical NEXT activit*) OR (physical NEXT performanc*) OR [mh "Physical Exertion"] OR "physical exertion" OR [mh "Physical Endurance"] OR (physical NEAR/4 endurance) OR "endurance training" OR (physical NEXT intensit*) OR [mh ^"Recreation"] OR (recreational NEXT activit*) OR [mh "Sports"] OR sport* OR stretching OR [mh "Yoga"] OR yoga OR [mh "Tai Ji"] OR "Tai Ji" OR "Tai Chi" OR (muscle NEXT strength*) OR (muscle NEAR/4 strengthening) OR "resistance training" OR (strength NEAR/4 training) OR (muscle NEXT strength*) OR "weight training" OR weightlifting OR "weight lifting" OR [mh "Walking"] OR walk* OR gait OR jog OR jogging OR running OR swimming OR aerobic* OR exertion* OR isometric* OR (energy NEXT expenditure*) OR (energy NEXT balanc*) OR (energy NEXT intake*) OR circuit* OR ("high intensity" NEXT interval*) OR ("high intensity" NEXT intermittent*) OR "HIIT" OR (sprint NEXT interval*) OR (stair* NEAR/3 climbing) OR calisthenic* OR cycling* OR biking* OR [mh "Sedentary Behavior"] OR sedentary OR (physical NEXT inactivit*)):ti,ab,kw |
| #3 | Mortality & Recurrence | ([mh "Neoplasm Recurrence, Local"] OR [mh "Recurrence"] OR recurren* OR reoccurren* OR relaps* OR [mh "Neoplasms, Second Primary"] OR "secondary events" OR (second NEXT cancer*) OR ("second primary" NEXT cancer*) OR (secondary NEXT cancer*) OR ("secondary primary" NEXT cancer*) OR [mh "Disease Progression"] OR progression* OR [mh "Disease-Free Survival"] OR (cancer NEXT survivor*) OR (cancer NEAR/3 survival) OR (cause NEAR/3 death) OR "all cause" OR fatal* OR [mh "Mortality"] OR mortalit* OR [mh "Quality of Life"] OR "quality of life" OR "life quality" OR "HRQO"):ti,ab,kw |
| #4 |  | #1 AND #2 AND #3 |
| #5 | Limits & Filters | [mh "Editorial"] OR [mh "Comment"] OR commentary* OR [mh "News"] OR [mh "Letter"] OR [mh "Retracted publication"] OR [mh "Retraction of publication"] OR (retraction NEAR/2 publication*) OR [mh "Congress"] OR [mh "Consensus Development Conference"] OR (conference NEXT abstract*) OR (conference NEXT proceeding*) OR (conference NEXT paper*) OR (conference NEXT review*) OR symposium* OR [mh "Case Reports"] OR (case NEXT report*) OR [mh "Review"] OR [mh "Systematic Review"] OR [mh "Systematic Reviews as Topic"] OR "systematic review" OR [mh "Meta-Analysis"] OR [mh "Meta-Analysis as Topic"] OR "meta-analysis" OR "meta-analyses" OR [mh "Practice Guideline"] |
| #6 | Limits & Filters | ("retraction notice" OR "retracted publication" OR review OR protocol OR protocols OR guideline*):ti |
| #7 |  | (#5 OR #6) |
| #8 | Limits & Filters | #4 NOT #7" with Publication Year from 2003 to 2023, with Cochrane Library publication date Between Jan 2003 and Sep 2023, in Trials (Word variations have been searched) |

Database: Embase
Platform: Elsevier
Date Searched: 10/04/2024

Date Limits: 1/1/2003 – 10/04/2024

|  | Concept: | Search Strategy: |
| --- | --- | --- |
| #1 | Breast Cancer | 'breast cancer'/exp OR ((breast* NEAR/4 (cancer* OR neoplas* OR carcinoma* OR tumor* OR tumour* OR malignan* OR sarcoma* OR lymphoma*)):ab,ti) |
| #2 | Physical Activity | 'exercise'/exp OR 'kinesiotherapy'/exp OR 'fitness'/exp OR 'endurance'/exp OR 'recreation'/de OR 'sport'/exp OR 'tai chi'/exp OR 'walking'/exp OR 'sedentary lifestyle'/exp OR 'exercise*':ab,ti OR 'physical fitness':ab,ti OR 'physical activit*':ab,ti OR 'physical performanc*':ab,ti OR 'physical exertion':ab,ti OR ((physical NEAR/4 endurance):ab,ti) OR 'endurance training':ab,ti OR 'physical intensit*':ab,ti OR 'recreational activit*':ab,ti OR 'sport*':ab,ti OR 'stretching':ab,ti OR 'yoga':ab,ti OR 'tai ji':ab,ti OR 'tai chi':ab,ti OR ((muscle NEAR/4 strengthening):ab,ti) OR 'resistance training':ab,ti OR ((strength NEAR/4 training):ab,ti) OR 'muscle strength*':ab,ti OR 'weight training':ab,ti OR 'weightlifting':ab,ti OR 'weight lifting':ab,ti OR 'walk*':ab,ti OR 'gait':ab,ti OR 'jog':ab,ti OR 'jogging':ab,ti OR 'running':ab,ti OR 'swimming':ab,ti OR 'aerobic*':ab,ti OR 'exertion*':ab,ti OR 'isometric*':ab,ti OR 'energy expenditure*':ab,ti OR 'energy balanc*':ab,ti OR 'energy intake*':ab,ti OR 'circuit*':ab,ti OR 'high intensity interval*':ab,ti OR 'high intensity intermittent*':ab,ti OR 'hiit':ab,ti OR 'sprint interval*':ab,ti OR ((stair* NEAR/3 climbing):ab,ti) OR 'calisthenic*':ab,ti OR 'cycling*':ab,ti OR 'biking*':ab,ti OR 'sedentary':ab,ti OR 'physical inactivit*':ab,ti |
| #3 | Mortality & Recurrence | 'tumor recurrence'/exp OR 'recurrent disease'/exp OR 'second primary neoplasm'/exp OR 'disease exacerbation'/exp OR 'disease free survival'/exp OR 'mortality'/mj OR 'quality of life'/exp OR 'recurren*':ab,ti OR 'reoccurren*':ab,ti OR 'relaps*':ab,ti OR 'secondary events':ab,ti OR 'second cancer*':ab,ti OR 'second primary cancer*':ab,ti OR 'secondary cancer*':ab,ti OR 'secondary primary cancer*':ab,ti OR 'progression*':ab,ti OR 'cancer survivor*':ab,ti OR ((cancer NEAR/3 survival):ab,ti) OR ((cause NEAR/3 death):ab,ti) OR 'all cause':ab,ti OR 'fatal*':ab,ti OR 'mortalit*':ab,ti OR 'quality of life':ab,ti OR 'life quality':ab,ti OR 'hrqol':ab,ti |
| #4 | Limits & Filters | #1 AND #2 AND #3 AND ([article]/lim OR [article in press]/lim)  AND [2003-2023]/py AND [english]/lim NOT ([animals]/lim NOT ([animals]/lim AND [humans]/lim)) NOT ('editorial'/exp OR 'letter'/exp OR 'note'/exp OR 'abstract report'/exp OR 'conference paper'/exp OR 'review'/exp OR 'systematic review'/exp OR 'systematic review (topic)'/exp OR 'meta analysis'/exp OR 'meta analysis (topic)'/exp OR 'case report'/exp OR 'practice guideline'/exp OR 'retraction notice'/exp OR 'retraction of publication':ab,ti OR 'retraction notice':ti OR 'retracted publication':ab,ti OR 'systematic review':ab,ti OR 'meta analysis':ab,ti OR 'meta analyses':ab,ti OR 'review':ti OR 'protocol':ti OR 'protocols':ti OR 'guideline*':ti OR 'case report*':ab,ti OR [conference abstract]/lim OR [conference paper]/lim OR [conference review]/lim OR 'symposium*':ab,ti OR 'conference abstract*':ab,ti OR 'conference review*':ab,ti OR 'conference proceeding*':ab,ti OR 'conference paper*':ab,ti) |

Database: CINAHL Plus
Platform: EBSCO*host*
Date Searched: 10/04/2024

Date Limits: 1/1/2003 – 10/04/2024

|  | Concept: | Search Strategy: |
| --- | --- | --- |
| #S1 | Breast Cancer | (MH "Breast Neoplasms+") OR TI ( (((MH "Breast+") OR breast*) AND (cancer* OR neoplas* OR carcinoma* OR tumor* OR tumour* OR malignan* OR sarcoma* OR lymphoma*)) ) OR AB ( (((MH "Breast+") OR breast*) AND (cancer* OR neoplas* OR carcinoma* OR tumor* OR tumour* OR malignan* OR sarcoma* OR lymphoma*)) ) |
| #S2 | Physical Activity | ((MH "Exercise+") OR (MH "Therapeutic Exercise+") OR (MH "Physical Fitness+") OR (MH "Exertion+") OR (MH "Physical Endurance+") OR (MH "Recreation") OR (MH "Sports+") OR (MH "Yoga+") OR (MH "Tai Chi") OR (MH "Walking+") OR (MH "Life Style, Sedentary+") ) OR TI ( exercise* OR "physical fitness" OR "physical activit*" OR "physical performanc*" OR "physical exertion" OR (physical W4 endurance) OR "endurance training" OR "physical intensit*" OR "recreational activit*" OR "sport*" OR "stretching" OR "yoga" OR "Tai Ji" OR "Tai Chi" OR "muscle strength*" OR (muscle W4 strengthening") OR "resistance training" OR (strength W4 training") OR "muscle strength*" OR "weight training" OR "weightlifting" OR "weight lifting" OR "walk*" OR "gait" OR "jog" OR "jogging" OR "running" OR "swimming" OR "aerobic*" OR "exertion*" OR "isometric*" OR "energy expenditure*" OR "energy balanc*" OR "energy intake*" OR "circuit*" OR "high intensity interval*" OR "high intensity intermittent*" OR "HIIT" OR "sprint interval*" OR (stair* W3 climbing) OR calisthenic* OR cycling* OR "biking* OR sedentary OR "physical inactivit*" ) OR AB ( exercise* OR "physical fitness" OR "physical activit*" OR "physical performanc*" OR "physical exertion" OR (physical W4 endurance) OR "endurance training" OR "physical intensit*" OR "recreational activit*" OR "sport*" OR "stretching" OR "yoga" OR "Tai Ji" OR "Tai Chi" OR "muscle strength*" OR (muscle W4 strengthening") OR "resistance training" OR (strength W4 training") OR "muscle strength*" OR "weight training" OR "weightlifting" OR "weight lifting" OR "walk*" OR "gait" OR "jog" OR "jogging" OR "running" OR "swimming" OR "aerobic*" OR "exertion*" OR "isometric*" OR "energy expenditure*" OR "energy balanc*" OR "energy intake*" OR "circuit*" OR "high intensity interval*" OR "high intensity intermittent*" OR "HIIT" OR "sprint interval*" OR (stair* W3 climbing) OR calisthenic* OR cycling* OR "biking* OR sedentary OR "physical inactivit*") |
| #S3 | Mortality & Recurrence | ( (MH "Neoplasm Recurrence, Local") OR (MH "Recurrence+") OR (MH "Neoplasms, Second Primary") OR (MH "Disease Progression+") OR (MH "Disease-Free Survival") OR (MH "Mortality+") OR (MH "Quality of Life+") ) OR TI ( recurren* OR reoccurren* OR relaps* OR "secondary events" OR "second cancer*" OR "second primary cancer*" OR "secondary cancer*"OR "secondary primary cancer*" OR progression* OR "cancer survivor*" OR (cancer N/3 survival) OR (cause N/3 death) OR "all cause" OR fatal* OR mortalit* OR "quality of life" OR "life quality" OR "HRQOL" ) OR AB ( recurren* OR reoccurren* OR relaps* OR "secondary events" OR "second cancer*" OR "second primary cancer*" OR "secondary cancer*"OR "secondary primary cancer*" OR progression* OR "cancer survivor*" OR (cancer N/3 survival) OR (cause N/3 death) OR "all cause" OR fatal* OR mortalit* OR "quality of life" OR "life quality" OR "HRQOL" ) |
| #S4 | Limits & Filters | #S4 NOT ( (((MH "Animals+") OR (MH "Animal Studies") OR (TI "animal model*")) NOT (MH "human")) ) NOT ( (MH "Congresses and Conferences") OR (MH "Edit and Review+") OR (MH "News") OR (MH "Literature Review+") OR (MH "Meta Analysis") OR (MH "Systematic Review") OR (MH "Retracted Publication") OR (MH "Retraction of Publication) OR (MH "Case Studies") OR (MH "Practice Guidelines") ) OR ( "conference abstract*" OR "conference proceeding*" OR "conference paper*" OR "conference review*" OR "symposium*" OR "case report*" OR "systematic review" OR "meta-analysis" OR "meta-analyses" OR "retraction of publication*" ) OR TI ( review OR protocol OR protocols OR "retraction notice" OR "retracted publication" OR guideline* ) Limiters - Publication Year: 2003-2023; English Language; Peer Reviewed Expanders - Apply equivalent subjects Search modes - Boolean/Phrase |

Database: PsycInfo
Platform: American Psychological Association
Date Searched: 10/04/2024

Date Limits: 1/1/2003 – 10/04/2024

|  | Concept: | Search Strategy: |
| --- | --- | --- |
| #1 | Breast Cancer | Index Terms: {Breast Neoplasms} *OR* Title: (((76) OR breast*) AND (cancer* OR neoplas* OR carcinoma* OR tumor* OR tumour* OR malignan* OR sarcoma* OR lymphoma*)) *OR* Abstract: (((76) OR breast*) AND (cancer* OR neoplas* OR carcinoma* OR tumor* OR tumour* OR malignan* OR sarcoma* OR lymphoma*)) |
| #2 | Physical Activity | Index Terms: {Exercise Therapy} OR {Exercise Therapy} OR {Physical Fitness} OR {Physical Endurance} OR {Recreation} OR (77) OR {Yoga} OR {Walking} OR {Sedentary Behavior} *OR* Title: exercise* OR "physical fitness" OR "physical activit*" OR "physical performanc*" OR "physical exertion" OR (physical NEAR/4 endurance) OR "endurance training" OR "physical intensit*" OR "recreational activit*" OR "sport*" OR "stretching" OR "yoga" OR "Tai Ji" OR "Tai Chi" OR "muscle strength*" OR (muscle NEAR/4 strengthening") OR "resistance training" OR (strength NEAR/4 training") OR "muscle strength*" OR "weight training" OR "weightlifting" OR "weight lifting" OR "walk*" OR "gait" OR "jog" OR "jogging" OR "running" OR "swimming" OR "aerobic*" OR "exertion*" OR "isometric*" OR "energy expenditure*" OR "energy balanc*" OR "energy intake*" OR "circuit*" OR "high intensity interval*" OR "high intensity intermittent*" OR "HIIT" OR "sprint interval*" OR (stair* NEAR/3 climbing) OR calisthenic* OR cycling* OR "biking* OR sedentary OR "physical inactivit*" *OR* Abstract: exercise* OR "physical fitness" OR "physical activit*" OR "physical performanc*" OR "physical exertion" OR (physical NEAR/4 endurance) OR "endurance training" OR "physical intensit*" OR "recreational activit*" OR "sport*" OR "stretching" OR "yoga" OR "Tai Ji" OR "Tai Chi" OR "muscle strength*" OR (muscle NEAR/4 strengthening") OR "resistance training" OR (strength NEAR/4 training") OR "muscle strength*" OR "weight training" OR "weightlifting" OR "weight lifting" OR "walk*" OR "gait" OR "jog" OR "jogging" OR "running" OR "swimming" OR "aerobic*" OR "exertion*" OR "isometric*" OR "energy expenditure*" OR "energy balanc*" OR "energy intake*" OR "circuit*" OR "high intensity interval*" OR "high intensity intermittent*" OR "HIIT" OR "sprint interval*" OR (stair* NEAR/3 climbing) OR calisthenic* OR cycling* OR "biking* OR sedentary OR "physical inactivit*" |
| #3 | Mortality & Recurrence | Index Terms: {Relapse (Disorders)} OR {Disease Progression} OR {Death and Dying} OR {Quality of Life} OR Title: recurren* OR Title: reoccurren* OR Title: relaps* OR Title: "secondary events" OR Title: "second cancer*" OR Title: "second primary cancer*" OR Title: "secondary cancer*" OR "secondary primary cancer*" OR Title: progression* OR Title: "cancer survivor*" OR (Title: cancer NEAR/3 survival) OR (Title: cause NEAR/3 death) OR Title: "all cause" OR Title: fatal* OR Title: mortalit* OR Title: "quality of life" OR Title: "life quality" OR Title: "HRQOL" OR Abstract: recurren* OR Abstract: reoccurren* OR Abstract: relaps* OR Abstract: "secondary events" OR Abstract: "second cancer*" OR Abstract: "second primary cancer*" OR Abstract: "secondary cancer*" OR "secondary primary cancer*" OR Abstract: progression* OR Abstract: "cancer survivor*" OR (Abstract: cancer NEAR/3 survival) OR (Abstract: cause NEAR/3 death) OR Abstract: "all cause" OR Abstract: fatal* OR Abstract: mortalit* OR Abstract: "quality of life" OR Abstract: "life quality" OR Abstract: "HRQOL" |
| #4 | Limits & Filters | ((IndexTermsFilt: ("Breast Neoplasms")) OR (Any Field: TitleFilt: ((("Breast" OR breast*)) AND TitleFilt: ((cancer* OR neoplas* OR carcinoma* OR tumor* OR tumour* OR malignan* OR sarcoma* OR lymphoma*)))) OR (Any Field: AbstractFilt: ((("Breast" OR breast*)) AND AbstractFilt: ((cancer* OR neoplas* OR carcinoma* OR tumor* OR tumour* OR malignan* OR sarcoma* OR lymphoma*))))) AND ((IndexTermsFilt: ("Exercise") OR IndexTermsFilt: ("Exercise Therapy") OR IndexTermsFilt: ("Physical Fitness") OR IndexTermsFilt: ("Physical Endurance") OR IndexTermsFilt: ("Recreation") OR IndexTermsFilt: ("Sports") OR IndexTermsFilt: ("Yoga") OR IndexTermsFilt: ("Walking") OR IndexTermsFilt: ("Sedentary Behavior")) OR title: (exercise* OR "physical fitness" OR "physical activit*" OR "physical performanc*" OR "physical exertion" OR (physical NEAR/4 endurance) OR "endurance training" OR "physical intensit*" OR "recreational activit*" OR "sport*" OR "stretching" OR "yoga" OR "Tai Ji" OR "Tai Chi" OR "muscle strength*" OR (muscle NEAR/4 strengthening ") OR " resistance training " OR (strength NEAR/4 training") OR "muscle strength*" OR "weight training" OR "weightlifting" OR "weight lifting" OR "walk*" OR "gait" OR "jog" OR "jogging" OR "running" OR "swimming" OR "aerobic*" OR "exertion*" OR "isometric*" OR "energy expenditure*" OR "energy balanc*" OR "energy intake*" OR "circuit*" OR "high intensity interval*" OR "high intensity intermittent*" OR "HIIT" OR "sprint interval*" OR (stair* NEAR/3 climbing) OR calisthenic* OR cycling* OR "biking* OR sedentary OR " physical inactivit* ") OR Abstract:(exercise* OR " physical fitness " OR " physical activit* " OR " physical performanc* " OR " physical exertion " OR (physical NEAR/4 endurance) OR " endurance training " OR " physical intensit* " OR " recreational activit* " OR " sport* " OR " stretching " OR " yoga " OR " Tai Ji " OR " Tai Chi " OR " muscle strength* " OR (muscle NEAR/4 strengthening") OR Any Field: "resistance training" OR (strength NEAR/4 training ") OR " muscle strength* " OR " weight training " OR " weightlifting " OR " weight lifting " OR " walk* " OR " gait " OR " jog " OR " jogging " OR " running " OR " swimming " OR " aerobic* " OR " exertion* " OR " isometric* " OR " energy expenditure* " OR " energy balanc* " OR " energy intake* " OR " circuit* " OR " high intensity interval* " OR " high intensity intermittent* " OR " HIIT " OR " sprint interval* " OR (stair* NEAR/3 climbing) OR calisthenic* OR cycling* OR " biking* OR sedentary OR "physical inactivit*")) AND ((IndexTermsFilt: ("Relapse (Disorders)") OR IndexTermsFilt: ("Disease Progression") OR IndexTermsFilt: ("Death and Dying") OR IndexTermsFilt: ("Quality of Life")) OR (title: (recurren*) OR title: (reoccurren*) OR title: (relaps*) OR title: ("secondary events") OR title: ("second cancer*") OR title: ("second primary cancer*") OR title: ("secondary cancer*" OR "secondary primary cancer*") OR title: (progression*) OR title: ("cancer survivor*") OR (title: (cancer NEAR/3 survival)) OR (title: (cause NEAR/3 death)) OR title: ("all cause") OR title: (fatal*) OR title: (mortalit*) OR title: ("quality of life") OR title: ("life quality") OR title: ("HRQOL")) OR (abstract: (recurren*) OR abstract: (reoccurren*) OR abstract: (relaps*) OR abstract: ("secondary events") OR abstract: ("second cancer*") OR abstract: ("second primary cancer*") OR abstract: ("secondary cancer*" OR "secondary primary cancer*") OR abstract: (progression*) OR abstract: ("cancer survivor*") OR (abstract: (cancer NEAR/3 survival)) OR (abstract: (cause NEAR/3 death)) OR abstract: ("all cause") OR abstract: (fatal*) OR abstract: (mortalit*) OR abstract: ("quality of life") OR abstract: ("life quality") OR abstract: ("HRQOL"))) AND Language: English AND NOT Population Group: Animal NOT Document Type: Abstract Collection OR Column/Opinion OR Comment/Reply OR Dissertation OR Editorial OR Letter OR Retraction OR Review-Book AND Peer-Reviewed Journals only AND Year: 2003 To 2023 |

Database: Web of Science (Core Collection)
Platform: Clarivate Analytics
Date Searched: 10/04/2024

Date Limits: 1/1/2003 – 10/04/2024

|  | Concept: | Search Strategy: |
| --- | --- | --- |
| #1 | Breast Cancer | TS=(breast* AND (cancer* OR neoplas* OR carcinoma* OR tumor* OR tumour* OR malignan* OR sarcoma* OR lymphoma*)) |
| #2 | Physical Activity | TS=(exercise* OR "physical fitness" OR "physical activit*" OR "physical performanc*" OR "physical exertion" OR (physical* NEAR/4 endurance*) OR "endurance training" OR "physical intensit*" OR "recreational activit*" OR sport* OR stretching OR yoga OR "Tai Ji" OR "Tai Chi" OR "muscle strength*" OR (muscle NEAR/4 strengthening) OR "resistance training" OR (strength NEAR/4 training) OR "muscle strength*" OR "weight training" OR "weightlifting" OR "weight lifting" OR walk*OR gait OR jog OR jogging OR running OR swimming OR aerobic* OR exertion* OR isometric* OR "energy expenditure*" OR "energy balanc*" OR "energy intake*" OR circuit* OR "high intensity interval*" OR "high intensity intermittent*" OR HIIT OR "sprint interval*" OR (stair NEAR/3 climbing) OR calisthenic* OR cycling* OR biking* OR sedentary OR "physical inactivit*") |
| #3 | Mortality & Recurrence | TS=(recurren* OR reoccurren* OR relaps* OR "secondary events" OR "second cancer*" OR "second primary cancer*" OR "secondary cancer*" OR "secondary primary cancer*" OR "progression*" OR "cancer survivor*" OR (cancer NEAR/3 survival) OR (cause NEAR/3 death) OR "all cause" OR fatal* OR mortalit* OR "quality of life" OR "life quality" OR HRQOL) |
| #4 | Limits & Filters | #3 AND #2 AND #1 NOT TS=(Commentary OR "retraction of publication*" OR "conference abstract*" OR "conference proceeding*" OR "conference paper*" OR "conference review*" OR "symposium*" OR "case report*" OR "systematic review" OR "meta-analysis" OR "meta-analyses")) NOT TI=(review OR "retraction notice" OR "retracted publication" OR protocol OR protocols OR guideline*) and Preprint Citation Index (Exclude – Database) and Clinical Trial or Other or Review Article or Clinical Trial or Abstract or Meeting or Dissertation Thesis or Data Set or Editorial Material or Book or Unspecified or Patent or Case Report or Data Study or Letter or News or Correction or Reference Material or Retracted Publication or Data Paper or Biography (Exclude – Document Types) and Animals or Disease Models Animal or Mice Inbred Balb C or Mice Nude or Mice or Cell Movement (Exclude – MeSH Headings) and English (Languages) and Web of Science Core Collection (Database) \| Timespan: 2003-01-01 to 2023-09-18 (Publication Date) |

Database: Scopus
Platform: Elsevier

Date Searched: 10/04/2024

Date Limits: 1/1/2003 – 10/04/2024

|  | Concept: | Search Strategy: |
| --- | --- | --- |
| #1 | Breast Cancer | TITLE-ABS-KEY(breast* AND (cancer* OR neoplas* OR carcinoma* OR tumor* OR tumour* OR malignan* OR sarcoma* OR lymphoma*)) |
| #2 | Physical Activity | TITLE-ABS-KEY(exercise* OR "physical fitness" OR "physical activit*" OR "physical performanc*" OR "physical exertion" OR (physical* W/4 endurance*) OR "endurance training" OR "physical intensit*" OR "recreational activit*" OR sport* OR stretching OR yoga OR "Tai Ji" OR "Tai Chi" OR "muscle strength*" OR (muscle W/4 strengthening) OR "resistance training" OR (strength W/4 training) OR "muscle strength*" OR "weight training" OR "weightlifting" OR "weight lifting" OR walk*OR gait OR jog OR jogging OR running OR swimming OR aerobic* OR exertion* OR isometric* OR "energy expenditure*" OR "energy balanc*" OR "energy intake*" OR circuit* OR "high intensity interval*" OR "high intensity intermittent*" OR HIIT OR "sprint interval*" OR (stair W/3 climbing) OR calisthenic* OR cycling* OR biking* OR sedentary OR "physical inactivit*") |
| #3 | Mortality & Recurrence | TITLE-ABS-KEY(recurren* OR reoccurren* OR relaps* OR "secondary events" OR "second cancer*" OR "second primary cancer*" OR "secondary cancer*" OR "secondary primary cancer*" OR "progression*" OR "cancer survivor*" OR (cancer W/3 survival) OR (cause W/3 death) OR "all cause" OR fatal* OR mortalit* OR "quality of life" OR "life quality" OR HRQOL) |
| #4 | Limits & Filters | (TITLE-ABS-KEY((breast* AND (cancer* OR neoplas* OR carcinoma* OR tumor* OR tumour* OR malignan* OR sarcoma* OR lymphoma*)))) AND (TITLE-ABS-KEY(exercise* OR "physical fitness" OR "physical activit*" OR "physical performanc*" OR "physical exertion" OR (physical* W/4 endurance*) OR "endurance training" OR "physical intensit*" OR "recreational activit*" OR sport* OR stretching OR yoga OR "Tai Ji" OR "Tai Chi" OR "muscle strength*" OR (muscle W/4 strengthening) OR "resistance training" OR (strength W/4 training) OR "muscle strength*" OR "weight training" OR "weightlifting" OR "weight lifting" OR walk*OR gait OR jog OR jogging OR running OR swimming OR aerobic* OR exertion* OR isometric* OR "energy expenditure*" OR "energy balanc*" OR "energy intake*" OR circuit* OR "high intensity interval*" OR "high intensity intermittent*" OR HIIT OR "sprint interval*" OR (stair W/3 climbing) OR calisthenic* OR cycling* OR biking* OR sedentary OR "physical inactivit*")) AND (TITLE-ABS-KEY(recurren* OR reoccurren* OR relaps* OR "secondary events" OR "second cancer*" OR "second primary cancer*" OR "secondary cancer*" OR "secondary primary cancer*" OR "progression*" OR "cancer survivor*" OR (cancer W/3 survival) OR (cause W/3 death) OR "all cause" OR fatal* OR mortalit* OR "quality of life" OR "life quality" OR HRQOL)) AND NOT INDEXTERMS (animal OR animals) AND NOT ((TITLE-ABS-KEY(Commentary OR "retraction of publication*" OR "conference abstract*" OR "conference proceeding*" OR "conference paper*" OR "conference review*" OR "symposium*" OR "case report*" OR "systematic review" OR "meta-analysis" OR "meta-analyses") OR TITLE(review OR "retraction notice" OR "retracted publication" OR protocol OR protocols OR guideline*))) AND PUBYEAR > 2002 AND PUBYEAR < 2024 AND (EXCLUDE (DOCTYPE,"re") OR EXCLUDE (DOCTYPE,"ch") OR EXCLUDE (DOCTYPE,"no") OR EXCLUDE (DOCTYPE,"ed") OR EXCLUDE (DOCTYPE,"le") OR EXCLUDE (DOCTYPE,"cp") OR EXCLUDE (DOCTYPE,"bk") OR EXCLUDE (DOCTYPE,"cr")) |

**Supplement 3. Inclusion and exclusion criteria**

| **Criteria** | **Inclusion** | **Exclusion** |
| --- | --- | --- |
| Date | - Published 2003 to present | - Published prior to 2003 |
| Population | - Breast cancer survivors | - Non-human subjects - Non-breast cancer survivors |
| Physical activity | - Measure post-diagnosis recreational and/or leisure-time aerobic physical activity in a way from which the proportion meeting aerobic physical activity recommendations could be determined | - Do not measure post-diagnosis physical activity (i.e. focus on pre-diagnosis physical activity) - Measure total physical activity or other domains (e.g. occupational physical activity), or focus specifically on sports - Combine aerobic and muscle-strengthening together into single variable - Use a poor-quality physical activity measure (e.g. any vs. none) |
| Concept | - Studies that include cardiovascular health (cardiovascular diseases, cardiac function, and/or related physiological risk factors) as an outcome | - The absence of outcomes stated within inclusion criteria |
| Type of Evidence | - Primary observational empirical research studies (e.g., cohort studies and cross-sectional studies) available in full text | - Trials/interventions - Reviews and meta-analyses - Editorials (e.g., perspectives, commentaries) - Abstracts, conference proceedings or posters - Dissertations/theses - Research protocols - Case reports - Patents - Articles for which the full text cannot be obtained |
| Language | - English | - Not available in English |

**Supplement 4. Quality appraisal**

| **Authors** | **Study/data source** | **Data Collection Method** | **S1** | **S2** | **3.1** | **3.2** | **3.3** | **3.4** |
| --- | --- | --- | --- | --- | --- | --- | --- | --- |
| Peck et al. 2022 (50) | EMBRACE-MRI 1 |  | ✓ | ✓ | ✓ | ✓ | X | X |
| Bao et al. 2013 (51) | SBCSS | Surveys, medical chart review, interview, physical examination | ✓ | ✓ | ✓ | ✓ | ✓ | ✓ |
| Obi et al. 2014 (52) | MARIE | Interviews, medical chart review | ✓ | ✓ | ✓ | ✓ | X | ✓ |
| Guinan et al. 2013(53) | Oncology clinics | Physical examination, survey | ✓ | ✓ | ✓ | X | ✓ | X |
| Kim et al. 2021 (48) | Korea NHIS | Medical chart review, surveys | ✓ | ✓ | ✓ | ✓ | ✓ | ✓ |
| Kim, So, & Kim, 2020 (49) | KHANES | Survey | ✓ | ✓ | ✓ | X | ✓ | X |
| Ariza-García et al. 2013 (54) | Oncology department | Medical chart review, surveys, physical examination | ✓ | ✓ | ✓ | X | ✓ | X |
| Nilsson et al. 2016 (55) | Hospitals | Surveys | ✓ | ✓ | ✓ | ✓ | ✓ | X |
| Busen et al. 2023 (46) | AABL | Surveys, interviews | ✓ | ✓ | ✓ | ✓ | ✓ | ✓ |
| Upshaw et al. 2020 (47) | CCT cohort | Medical chart review, surveys, physical examination | ✓ | ✓ | ✓ | ✓ | ✓ | ✓ |
| Irwin et al. 2005 (42) | HEAL | Interview, physical examination, survey | ✓ | ✓ | ✓ | ✓ | X | ✓ |
| Jones et al. 2016 (43) | LACE + Pathways | Surveys, medical chart review | ✓ | ✓ | ✓ | ✓ | X | ✓ |
| Marell et al. 2023 (44) | Breast Disease Registry | Surveys | ✓ | ✓ | ✓ | X | X | X |
| Dieli-Conwright et al. 2022 (45) | WHI | Surveys, physical examination, medical chart review | ✓ | ✓ | ✓ | ✓ | ✓ | ✓ |

Notes. S1: Are research question(s) clear?; S2: Is data collection appropriate to answer research question(s)?; 3.1: Are participants representative of target population?; 3.2: Are measurements appropriate?; 3.3: Is outcome data complete?; 3.4 Are confounders accounted for in design or analysis?; ✓ = Yes; X = No; AABL = African American Breast Cancer Long-Term Survivorship; CCT = Cardiotoxicity of Cancer Therapy; EMBRACE-MRI 1 = Evaluation of Myocardial Changes During Breast Adenocarcinoma Therapy to Detect Cardiotoxicity Earlier With MRI; HEAL = Health, Eating, Activity, and Lifestyle; KHANES = Korea National Health and Nutrition Examination Survey; LACE = Life After Cancer Epidemiology; MARIE = Mamma carcinoma Risk factor Investigation; NHIS = National Health Insurance Service; SBCSS = Shanghai Breast Cancer Survival Study; WHI = Women’s Health Initiative

**Supplement 5. Aerobic exercise measurement and guidelines**

| **Authors** | **Study/data source** | **Measure** | **Guidelines (Organization)** | **Proportion meeting**  **guidelines (%)** |
| --- | --- | --- | --- | --- |
| Peck et al. 2022 (50) | EMBRACE-MRI 1 | GLTEQ(78) | ≥90min MVPA/week (ACSM)(18) | 47.7% |
| Bao et al. 2013 (51) | SBCSS | SWHS PAQ (leisure-time)(79) | >3.5hrs/week(80) | 46.5% |
| Obi et al. 2014 (52) | MARIE | Unnamed (recreational)(81) | - | - |
| Guinan et al. 2013 (53) | Oncology clinics | MLTPAQ(82) | ≥30 min of MPA, ≥5 days/week (ACSM)(57) | 24.6% |
| Kim et al. 2021 (48) | Korea NHIS | IPAQ-LF (recreational)(83) | ≥500 MET-min/week (uncited) | 53.3% |
| Kim, So, & Kim, 2020 (49) | KHANES | Unclear (recreational) | ≥150 min/week of MPA or ≥75 min/week of VPA | 43.0% |
| Ariza-García et al. 2013 (54) | Oncology department | MLTPAQ(84) | ≥3 MET-hrs/week | 63.4% ‘active’ |
| Nilsson et al. 2016 (55) | Hospitals | SGPALS | - | - |
| Busen et al. 2023 (46) | AABL | AAFQ (recreational) | ≥150 min/week of MPA or ≥75 min/week of VPA (recreational) (ACS, USDHHS)(19, 58) | 39.6% |
| Upshaw et al. 2020 (47) | CCT cohort | GLTEQ(85) | ~150 min/week of MVPA | 12.1% |
| Irwin et al. 2005(42) | HEAL | MAQ (sports and recreation)(56) | - | - |
| Jones et al. 2016 (43) | LACE + Pathways | AAFQ (recreational)(86, 87) | 3-5 sessions of MVPA/week of ≥20 minutes, the equivalent of ≥9 MET-hrs/week(88) | 53.2% |
| Marell et al. 2023 (44) | Mayo Clinic Breast Disease Registry | GLTEQ(89) | ≥150 min/week of MVPA (ACSM, USDHHS, NCCN)(18, 19, 59) | Baseline - 39.2%; 1 year - 39.8%; 4 years - 36.3% |
| Dieli-Conwright et al. 2022 (45) | WHI | WHI-PAQ (recreational)(90) | ≥9 MET-hrs/week | 43.4% |

Notes. AABL = African American Breast Cancer Long-Term Survivorship; AAFQ = Arizona Activity Frequency Questionnaire; ACS = American Cancer Society; ACSM = American College of Sports Medicine; CCT = Cardiotoxicity of Cancer Therapy; EMBRACE-MRI 1 = Evaluation of Myocardial Changes During Breast Adenocarcinoma Therapy to Detect Cardiotoxicity Earlier With MRI; GLETQ = Godin Leisure Time Exercise Questionnaire; HEAL = Health, Eating, Activity, and Lifestyle; IPAQ-LF = International Physical Activity Questionnaire Long Form; KHANES = Korea National Health and Nutrition Examination Survey; LACE = Life After Cancer Epidemiology; MARIE = Mamma carcinoma Risk factor Investigation; MET = Metabolic equivalent; MLTPAQ = Minnesota Leisure Time Physical Activity Questionnaire; MAQ = Modifiable Activity Questionnaire; NCCN = National Comprehensive Cancer Network; NHIS = National Health Insurance Service; SGPALS = Saltin-Grimby Physical Activity Level Scale; SWHS PAQ = Shanghai Women’s Health Study Physical Activity Questionnaire; USDHHS = U.S. Department of Health and Human Services; WHI = Women’s Health Initiative; WHI-PAQ = WHI Physical Activity Questionnaire; MPA = Moderate intensity physical activity; VPA = Vigorous intensity physical activity; MVPA = Moderate-to-vigorous intensity physical activity

**Supplement 6. Associations of exercise with cholesterol related outcomes**

| **Study** | **Cholesterol outcomes** | **Findings** | | |
| --- | --- | --- | --- | --- |
| **Adjusted analyses** | | | | |
| Bao et al. 2013 (51)** | High-density lipoprotein cholesterol (<1.3 mmol/l) | Referent: No exercise <3.5hrs/week: OR:1.10 (95%CI:0.79-1.53) ≥3.5hrs/week: OR:1.01 (95%CI:0.73-1.40) | | |
| Upshaw et al. 2020 (47)*** | Hyperlipidemia*^+clinical chart review^ | β = -2.81 (95%CI:-5.04-0.59) | | |
| **Unadjusted analyses** | | | | |
|  | | **Exercise** | | |
|  |  | **Guidelines** | **Met guidelines** | |
|  |  |  | **Yes** | **No** |
| Busen et al. 2023 (46) | High cholesterol* | ≥150 min/week of MPA or ≥75 min/week of VPA (ACS/USDHHS) | 36.7% | 46.2% |
| Dieli-Conwright et al. 2022 (45) | History of high cholesterol* | ≥9 MET-hrs/week | 13.2% | 13.9% |
| Jones et al. 2016 (43) | Hyperlipidemia | ≥9 MET-hrs/week | 23.8% | 27.5% |
| Kim et al. 2021 (48) | Total cholesterol (mg/dL) | >500 MET-mins/week | M (SD) | |
|  |  |  | 193.8 (38.3) | 196.1 (39.8) |
| Marell et al. 2023 (44) | Hypercholesteremia* | ≥150 min/week of MVPA (ACSM, USDHHS, NCCN) | 25.4% | 39.9% |

Notes. * = Self-report; **Adjusted for age at diagnosis, education, body mass index (BMI) at baseline, menopausal status at baseline, disease (Charlson comorbidity score 0/≥1), and Tumor, Node, Metastasis (TNM) stage; ***Not reported

ACS = American Cancer Society; ACSM = American College of Sports Medicine; MET = Metabolic equivalent; MPA = Moderate intensity physical activity; VPA = Vigorous intensity physical activity; MVPA = Moderate-to-vigorous intensity physical activity; NCCN = National Comprehensive Cancer Network; USDHHS = U.S. Department of Health and Human Services;
